# Mapping Export Sustainability in Frozen Cephalopods: Destination Concentration and Revealed Comparative Advantage in China, Indonesia, India, Spain, Morocco, and Argentina (2020–2024)

**DOI:** 10.12688/f1000research.183651.1

**Published:** 2026-06-18

**Authors:** Jose Carlos Montes Ninaquispe, Luisa Angelica Orejuela Guerrero, Eleodora del Pilar Orejuela Guerrero, Carlos José Sandoval Reyes, José Alfredo Castañeda Nassi, Victor Ángel Ancajima Miñán, Rosario Yaqueliny Llauce Santamaria, Juan Carlos Damián Sandoval, Karina Tomasa Valdiviezo Pérez, César Pol Arévalo-Aranda, Marco Agustín Arbulú Ballesteros

**Affiliations:** 1 Programa de Administración y Administración de Negocios Internacionales Filial Norte, Universidad de San Martín de Porres, Chiclayo, 14001, Peru; 2 Departamento de Ingeniería, Escuela Profesional de Ingeniería Industrial, Facultad de Ingeniería, Universidad Nacional de Trujillo, Trujillo, La Libertad, 13011, Peru; 3 Departamento de Ingeniería, Escuela Profesional de Ingeniería Ambiental, Facultad de Ingeniería, Universidad Privada del Norte, Campus San Isidro-Trujillo, Trujillo, La Libertad, 13001, Peru; 4 Escuela de Administración, Facultad de Ciencias Económicas, Universidad Privada Antenor Orrego, Trujillo, La Libertad, 13008, Peru; 5 Institute for Research in Science and Technology, César Vallejo University, Campus Chepén-Piura, Trujillo, La Libertad, 13001, Peru; 6 Departamento Académico de Ciencias Básicas y Aplicadas, Facultad de Ingeniería, Universidad Nacional de Jaén, Jaén, Cajamarca, 06801, Peru; 7 Departamento Académico de Ingeniería Metalúrgica, Escuela Profesional de Ingeniería Metalúrgica, Universidad Nacional de Trujillo, Trujillo, La Libertad, 13011, Peru

**Keywords:** export diversification; revealed comparative advantage; Herfindahl–Hirschman Index; frozen cephalopods; HS 030743; UN Comtrade; seafood trade; export concentration.

## Abstract

**Background:**

Frozen squid and cuttlefish (Harmonized System code 030743) form a commercially relevant segment of global cephalopod trade, with implications for cold-chain logistics, traceability and the economic resilience of exporting countries. Despite this relevance, comparable cross-country evidence on how export portfolios are structured and how relative specialization evolves remains limited. The study describes and compares destination diversification and revealed export positioning of frozen cephalopod exports for China, Indonesia, India, Spain, Morocco and Argentina between 2020 and 2024.

**Methods:**

A quantitative, non-experimental, retrospective longitudinal design was applied using secondary statistics from the United Nations International Trade Statistics Database (UN Comtrade). Destination diversification was measured through the Herfindahl–Hirschman Index (HHI), computed from the complete distribution of export shares across importing partners. Revealed specialization was assessed through the Revealed Symmetric Comparative Advantage (RSCA), obtained from the symmetric transformation of the Balassa index. Consistent with a strictly descriptive scope, no correlational or causal procedures were performed; cleaning and harmonization followed documented routines to ensure replicability.

**Results:**

Cross-country heterogeneity was marked. China showed sustained deconcentration, with HHI declining from 1,134 to 704. India maintained a stable, moderately concentrated portfolio. Argentina moved from high concentration toward a more distributed structure, with partial reconcentration in 2024. Indonesia deepened its concentration, with HHI rising from 2,964 to 3,894, and Morocco remained highly concentrated throughout. Spain preserved high concentration despite redistribution among secondary markets. RSCA values evidenced persistent revealed specialization for India and Argentina, China’s repositioning in selected destinations, Indonesia’s Asia-centered specialization, and Morocco’s competitiveness embedded in Mediterranean outlets.

**Conclusions:**

Three descriptive export profiles were identified: diversified and stable; growth-oriented yet dependent; and specialized but destination-vulnerable. This typology offers a replicable baseline for sustainability discussions without inferring causal mechanisms.

## 1. Introduction

Global exports of fisheries and aquaculture products have become strategically central to contemporary sustainability debates, not only because of their contribution to food security, but also because of their role in generating income, employment, and cross-border production linkages in increasingly globalized food systems. ^1^ In this context, cephalopods—particularly squid and cuttlefish—have consolidated their position as a resource group of high commercial and cultural relevance, with capture, processing, and trade dynamics that combine biological complexity (short life cycles and high interannual variability) with strong market pressures, regulatory demands, and traceability challenges. This intersection of living resources, global value chains, governance, and trade performance is especially relevant because foreign trade metrics are not merely economic indicators; they also operate as indirect signals of vulnerability, dependence, and resilience in production systems facing environmental, regulatory, and demand shocks.

Within the broader universe of cephalopod trade, this study focuses on product HS 030743 (frozen cuttlefish and squid), as this category captures two features that are crucial for the sustainability of modern fisheries chains: (i) the predominance of a frozen format, which typically implies cold-chain infrastructure, international logistics, standardization, and, in many cases, industrial processing; and (ii) the possibility of observing, through customs data, relatively comparable cross-country patterns in terms of destinations, relative shares, and positioning in the global market. In line with standards for replicable research in trade economics and sustainability, the analysis relies on international trade statistics and harmonized classifications, which are widely used in applied seafood research. ^2^

The empirical focus is on six countries—China, Indonesia, India, Spain, Morocco, and Argentina—selected because of their recurrent presence in academic and policy discussions related to cephalopod production, fishing fleets, processing capacity, trade, and governance. China occupies a prominent place in debates on ocean fisheries and fleets operating beyond national jurisdictions, particularly squid fisheries associated with light-attraction vessels, which has motivated studies highlighting regulatory gaps and monitoring challenges. ^3^ In the Southwest Atlantic, Argentina is an emblematic case due to the interaction between exclusive economic zones, high-seas activity, and squid-related geopolitical tensions, with direct implications for sustainability and governance. ^4^ Spain, in turn, is embedded in the European seafood consumption and import system, where demand, regulation, and market standards jointly shape incentives for traceability and compliance. ^5^ Morocco is linked to debates on the political and economic configuration of fisheries sectors along the Northwest African Atlantic coast and their export projection toward demanding markets, in contexts where sustainability is intertwined with institutional disputes and arrangements. ^6^ Finally, India and Indonesia are associated with supply-side dynamics involving extraction and processing capacity, fleet heterogeneity, and governance and control challenges in complex marine product chains, making it especially relevant to describe their export positioning without presuming untested causal mechanisms. ^7^ ^,^ ^8^

In terms of relevance, a diagnosis of destination diversification can help interpret exposure to risks arising from trade dependence, while the Revealed Symmetric Comparative Advantage (RSCA) allows a comparative assessment of the product’s relative export positioning against the global specialization pattern. ^9^ The contribution of this study, therefore, does not rest on causal inference, but on constructing an empirical and comparable “mapping” of how destinations are distributed and how each country’s relative positioning is expressed in the global market, as emphasized in studies that analyze these indicators. ^10^ ^–^ ^12^

This need for mapping becomes more pressing when one considers that the sustainability of seafood supply chains faces growing demands for transparency, traceability, and compliance, driven by both regulation and reputational and market pressures. ^13^ ^,^ ^14^ The literature on cephalopod traceability has warned that, compared with other groups (e.g., fish), monitoring and standardization mechanisms for cephalopods have developed unevenly, leaving persistent gaps in identification, documentation, and control along the chain. ^15^ Recent analyses also stress that “declared” traceability is not always equivalent to operational and verifiable traceability, and that current tools may be insufficient to sustain claims of sustainability and legality in global seafood chains. ^16^ In parallel, studies combining satellite data and empirical evidence have documented the coexistence of risks related to illegal, unreported, and unregulated fishing and labor abuses in certain fleet segments and regions, challenging any simplified sustainability narrative based solely on aggregate trade data. ^17^

In this context, international trade reflects not only market preferences or cost advantages, but also—often with lags and distortions—the outcomes of regulatory frameworks, control capacities, infrastructure, integration into global value chains, and quality standards. ^18^ This is particularly visible in squid fisheries: research on the underregulated nature of global squid fisheries has shown that, in the absence of robust governance arrangements, exploitation and supply dynamics can expand rapidly, driven by demand and technological capabilities for detection and capture. ^19^ At the macro-institutional level, attempts to realign incentives through trade and environmental disciplines (e.g., the Agreement on Fisheries Subsidies) further reinforce the importance of understanding how export specializations in marine products are configured and reconfigured. ^20^

In sum, the study is justified by three convergent reasons: (i) the commercial centrality of frozen cephalopods and the analytical value of observing them; (ii) the presence of six countries with significant profiles in the international trade of this product; and (iii) the need for comparable descriptive diagnoses that are useful for sustainability discussions without forcing causal relationships. Accordingly, the general objective is to describe and compare, for China, Indonesia, India, Spain, Morocco, and Argentina, (1) destination diversification (HHI) and (2) the symmetric RCA (RSCA) of HS 030743 exports using a replicable international trade framework. The guiding research question is: how are destination concentration/diversification and relative export positioning for HS 030743 characterized and how do they evolve, comparatively, across the six selected countries within the global market?

### 1.1. Literature review

Recent cephalopod literature converges on the idea that, while their commercial importance has increased, major structural sustainability challenges persist in relation to biology, exploitation, and governance. A key contribution has been the development of integrated assessments combining pressure, state, and institutional response to estimate the sustainability of cephalopod fisheries across multiple countries, highlighting important heterogeneity among economies and showing that response capacities (governance, control, and stressor mitigation) are decisive for limiting risk. ^21^ These differences matter for trade analysis because supply stability, compliance costs, and market acceptance may vary across countries and translate into shifts in destinations, relative prices, or specialization.

A second line of research emphasizes that cephalopod system sustainability cannot be addressed without considering biological uncertainty and methodological limits in stock assessment. Because many cephalopod fisheries operate under data-limited conditions, with high environmental variability and short life cycles, “rapid” assessment approaches and alternative methods have been proposed to support more timely decisions, underscoring that limited high-frequency data infrastructure is itself a sustainability problem. ^22^ ^,^ ^23^ For trade analysis, this suggests that abrupt changes in exportable volumes may coexist with apparent stability in annual aggregates, reinforcing the need for descriptive approaches that do not confuse ecological variation with “trade strategy.”

Evidence has also strengthened the link between cephalopods and climate change, albeit in complex and non-uniform ways. A study on climate change impacts on cephalopods reported consistent signs of ecological sensitivity and biogeographic responses, showing that variations in temperature and oceanographic conditions can alter distribution, abundance, and catch conditions at regional scales. ^24^ This line of research does not replace trade analysis, but it helps contextualize why export series may exhibit episodes of expansion or contraction that, descriptively, appear as changes in destination shares or relative participation without requiring immediate causal interpretation.

Fishing-related criminality has also been examined through global approaches that reveal concentration patterns in fisheries crimes and the ability to identify actors and structures, reinforcing the idea that sustainability requires monitoring and enforcement instruments beyond production and trade metrics. ^25^ Although the present study does not estimate effects, it suggests that changes in destinations or specialization may occur in contexts where governance and control are under strain, particularly in high-seas fisheries and distant-water fleets.

In the specific case of squid, the literature has advanced by documenting the magnitude of regulatory gaps in global fisheries associated with light-attraction fleets and transboundary operations. ^19^ One study argued that a substantial share of global activity takes place under weak or nonexistent regulation, raising major sustainability and international governance dilemmas. ^19^ This diagnosis is particularly relevant for countries such as China and, by extension, adjacent regions, where high-seas fishing pressure has become a recurring topic in public and academic debate. ^26^

At the regional and geopolitical scale, the evidence shows that high-seas squid fishing—especially near national jurisdictions—can generate tensions at the intersection of sustainability and sovereignty. ^27^ An analysis focused on Chinese fleets operating near South American EEZs described squid fishing as a phenomenon that combines resource pressure, governance gaps, and geopolitical frictions. ^4^ Such studies help explain why Argentina, for example, occupies a distinctive place in squid-related debates and why trade in squid-derived products may be shaped by narratives of sustainability, surveillance, and control.

Complementary research has examined opportunities and gaps in international law for addressing the underregulation of high-seas squid fisheries. A recent legal review identified partial progress alongside persistent normative gaps and proposed opportunities for more effective management. ^28^ This discussion is relevant for trade because regulatory evolution may eventually translate into new requirements for documentation, traceability, and compliance, thereby reshaping export destinations and processing incentives.

Alongside governance, another important field concerns product traceability and authenticity. Evidence suggests that cephalopod markets may be exposed to species substitution and mislabeling problems, especially in processed products or those with morphologies that are difficult to distinguish. ^29^ A recent study applied to cephalopod markets documented mislabeling rates and discussed implications for fraud control, food safety, and market governance. In frozen products, where processing may reduce morphological cues, traceability becomes even more critical.

Consistently, the literature has warned that cephalopod traceability lags behind that of other seafood groups and that chain-wide standardization (from capture to commercialization) remains uneven. ^30^ A synthesis study emphasized that progress toward sustainability requires better integration of species identification, origin documentation, stock monitoring, and traceability across global cephalopod chains. ^15^ This body of work reinforces the point that trade, even when legal and reported, may not by itself capture critical sustainability attributes unless supported by verification systems.

Another set of studies has examined the role of states in promoting traceability and sustainability in fisheries, showing that policy design may produce non-trivial effects, including the displacement of problems and the reconfiguration of incentives along the chain. A recent study on state-led traceability discussed mechanisms through which traceability may support sustainability, but also highlighted risks of “externalization” when formal compliance is emphasized without addressing structural asymmetries. ^31^ This is particularly relevant because high-standard markets (such as several European markets) may induce changes in export destinations and trade forms, but such changes should not be interpreted automatically as substantive improvements without additional evidence.

Regarding social sustainability, the literature has also stressed that labor justice analyses in seafood supply chains can become constrained by methodological nationalism, which underestimates the transnational structure of these chains and complicates attribution of responsibility and remedy. A recent contribution showed how labor justice analysis benefits from a more explicitly transnational perspective in export-oriented marine product chains. ^32^ This perspective again reinforces why a descriptive trade diagnosis should be understood as one component of a broader sustainability puzzle.

In the literature specifically focused on international cephalopod trade, important advances have been made in mapping global trade networks using reported trade data. An influential study provided a long-run overview of legal cephalopod trade, identifying dominant actors, centrality patterns, and market asymmetries. ^30^ This type of evidence supports the present study’s approach by showing that, even without causal modeling, descriptive analysis of customs data can reveal structures of dependence and concentration across countries and destinations.

Another study on cephalopod markets highlighted that the global trade network includes a very large number of countries, but is dominated by a relatively small group of economies—particularly in Asia—with interactions among demand, food security, control systems, and authenticity. ^33^ This supports the selection of focal countries such as China and India and strengthens the argument that destination diversification and relative specialization may vary substantially according to industrial structures and historical trade linkages.

Within the literature on trade networks and fisheries sustainability, recent studies have used network metrics to examine global patterns, emphasizing that trade configuration may be associated with different sustainability scenarios and relative biomass conditions, thereby informing debates on how the architecture of international trade may support—or strain—sustainability objectives. ^34^ Although the present study does not aim to test relationships, this literature justifies why describing concentration/diversification and export positioning is useful for sustainability audiences: these are indicators of trade architecture that may amplify or buffer risks.

In resource-specific studies, there has also been growing interest in the spatial dynamics and abundance of Argentine squid (Illex argentinus) in the South Atlantic, with forecasting methods integrating oceanographic variables. A recent study on spatiotemporal abundance in the Falkland/Malvinas area improved understanding of resource availability in a key squid-producing zone, which is relevant for interpreting supply fluctuations that may be reflected in frozen-product exports. ^35^ This evidence supports the idea that, even in purely trade-based analyses, ecological variability is an indispensable interpretive context.

Meanwhile, studies on artisanal cephalopod fisheries in Northwest Africa have documented sustainability tensions in settings where the resource is socially and economically crucial. A Marine Policy study on the artisanal octopus fishery in Mauritania reported signs of decline and discussed implications for management, employment, and income, providing a regional framework that resonates with sustainability concerns in nearby Atlantic cephalopod systems. ^36^ This suggests that trade flows in frozen cephalopods may coexist with local sustainability pressures in exporting regions.

In Asia, where a substantial share of global cephalopod activity is concentrated, the literature has examined ecological and operational dimensions of squid-oriented fisheries. A study on purpleback flying squid (Sthenoteuthis oualaniensis) in the northwestern Indian Ocean used nonlinear models and machine learning to explain distribution and catches, highlighting the relevance of temporal and thermal factors in fishery performance. ^37^ Although biological-operational in focus, this work helps contextualize supply dynamics for China and associated fishing areas.

Similarly, other studies have analyzed squid fishing zones and fleets in the Indian Ocean with implications for distant-water fisheries. A recent study investigated factors influencing the location of squid jigging activity in the northwestern Indian Ocean, offering empirical evidence on spatial patterns of fishing effort and catches. ^38^ By emphasizing spatial and temporal heterogeneity, this literature reinforces interpretive caution: changes in trade metrics may reflect both resource conditions and market or logistical reconfiguration.

In Europe, the literature on marine sustainability and biodiversity has incorporated cephalopods into discussions of ecosystem-based management and environmental policy frameworks. A Marine Biology study examined the role of cephalopods under the EU Marine Strategy Framework Directive, providing evidence on monitoring and conservation challenges with indirect implications for European countries linked to cephalopod capture and consumption. ^39^ For a country such as Spain, embedded in the European seafood market, these debates form part of the regulatory and sustainability environment in which import and export flows operate.

Complementarily, conservation literature has stressed that cephalopod biodiversity management is hindered by knowledge and monitoring gaps, which may bias status assessment and management policies. A recent synthesis identified knowledge gaps and challenges for cephalopod conservation and management in contexts where exploitation is economically important. ^40^ This evidence helps explain why cephalopod sustainability requires integrated approaches that go beyond purely trade-based indicators.

From a methodological standpoint, the trade-and-sustainability literature recommends caution when interpreting foreign trade indicators as direct proxies for sustainability. The reason is twofold: reported trade data do not fully capture informality, underreporting, or externalities, and marine product chains may undergo reconfiguration driven by standards, certifications, and policy changes. A recent review on export diversification and economic vulnerability in African monetary unions showed, from an applied perspective, that concentration/diversification is associated with exposure to external shocks, reinforcing the usefulness of concentration as a descriptive risk indicator. ^41^ Although the object of that study is macroeconomic, the argument is transferable: an export portfolio concentrated in a few destinations can amplify vulnerability in product chains.

Finally, to complete the relevant literature landscape, methodological contributions on specialization indicators and revealed comparative advantage are essential, since the symmetric RCA (RSCA) is widely used. One study discussed how the RCA distribution tends to be non-normal and reviewed transformations and alternative indices, which is relevant for justifying why even descriptive studies should explicitly state the “symmetric” nature of the indicator and its interpretation. ^9^ In addition, recent studies have assessed RCA across sectors and economies, showing its usefulness for describing changes in relative specialization over time. ^42^ This literature supports the use of symmetric RCA as a descriptive tool, especially when the objective is cross-country comparability without extrapolating causal conclusions.

In summary, the literature review supports five ideas that justify the study: (i) cephalopod sustainability is heterogeneous and involves integrated challenges related to ecological pressure, governance, and institutional response; (ii) squid fisheries in particular face major regulatory gaps on the high seas, with sustainability and geopolitical implications; (iii) cephalopod traceability and market authenticity remain areas with persistent gaps, especially relevant for frozen products ^29^; (iv) reported trade patterns can be mapped using network approaches and provide useful evidence for describing structures of dominance and dependence; and (v) concentration/diversification and relative specialization metrics, even without causal inference, are appropriate descriptive tools for discussing risk, resilience, and sustainability in global chains.

### 1.2. Theoretical framework

The theoretical framework of this study integrates three components: (i) the conceptualization of international seafood trade as part of socioecological systems and global value chains; (ii) destination diversification/concentration as a descriptive attribute of the export “portfolio”; and (iii) relative specialization measured through symmetric RCA (RSCA) as an indicator of export positioning in the global market. These components are articulated without postulating causal relationships between the two central indicators (HHI and RSCA), because the scope is strictly descriptive.

First, international trade in fishery products can be understood as an observable manifestation of socioeconomic networks linking extraction, processing, logistics, regulation, and consumption. In cephalopods, this network is characterized by broad geographic coverage and the dominance of certain nodes, with clear implications for sustainability and governance. ^33^ From this perspective, customs data make it possible to observe flows, destinations, and relative shares, although they do not by themselves reveal internal sustainability attributes (e.g., legality, stock status, or decent work conditions). Accordingly, the framework adopted here treats trade indicators as descriptive “layers” that must be interpreted within a broader sustainability perspective in which traceability and governance are enabling conditions for aligning trade flows with environmental and social objectives. ^16^

Second, destination diversification is theoretically grounded in the portfolio concept: an exporting country distributes its supply across multiple destination markets, and that distribution may be more concentrated or more diversified. ^43^ The Herfindahl–Hirschman Index (HHI) is widely used to capture this structure through the sum of squared destination shares within the product’s total exports. Conceptually, higher values indicate concentration (dependence on a few destinations), whereas lower values indicate a more balanced distribution across markets. From the standpoint of economic sustainability, this structure is linked to exposure to external shocks, regulatory changes, demand variation, or logistics disruptions, making it a useful descriptive risk diagnostic. ^41^ For a product such as HS 030743, where cold-chain logistics and sanitary compliance can condition market access, destination diversification may be interpreted as an attribute of commercial resilience, without inferring that such resilience derives from any specific cause.

In applied sustainability and trade studies, diversification and competitiveness have been proposed as complementary dimensions for discussing export sustainability, although empirical evidence and mechanisms vary by sector. A study published in *Sustainability* discusses export “sustainability” through diversification and competitiveness in comparative analyses, illustrating how these metrics can be used for characterization and diagnosis without assuming invariant relationships between indicators. ^44^ In the present study, that perspective is adopted only in its descriptive dimension: HHI is interpreted as the destination structure of HS 030743 by country and year.

Third, symmetric Revealed Comparative Advantage (RSCA) is grounded in the tradition of international specialization “revealed” through export patterns. In its classical form, RCA compares the share of a product in a country’s exports with the share of the same product in world exports. It is considered “asymmetric” because its scale is not centered or bounded symmetrically: values below 1 indicate revealed comparative disadvantage, while values above 1 indicate revealed comparative advantage, but the magnitude above 1 can increase without an upper bound equivalent to the lower bound. ^9^ This asymmetry has motivated extensive discussion on statistical properties, comparability, and alternative transformations, yet RCA remains attractive because of its simplicity and interpretability as an indicator of relative specialization.

Even so, in descriptive trade studies—especially in agri-food and natural resource products—RCA is typically used to trace specialization profiles by country and their temporal evolution, rather than to explain underlying causes. ^45^ Consistent with this use, the present study adopts symmetric RCA (RSCA) as an indicator of the relative positioning of HS 030743, reported by country and year, without using it as an explanatory variable for any other indicator.

Another relevant strand of literature examines the link between comparative advantage measured in gross exports and production fragmentation in global value chains, which may alter the interpretation of specialization when an economy participates in specific processing stages or re-export activities. A recent study, from a value-added trade perspective, proposed that comparative advantage can be analyzed using dynamic methodologies, emphasizing that interpretations change when value-added content is considered. ^46^ This discussion is pertinent for HS 030743 because frozen products may involve different degrees of processing and international linkages; therefore, the theoretical framework recognizes interpretive limits of RCA/RSCA in the presence of productive fragmentation, while the study itself remains at the descriptive level of recorded exports.

Finally, because the empirical object is HS 030743 exports, the framework assumes the validity of the “reported trade” approach as an approximation to recorded legal flows, while recognizing known limitations (underreporting, reclassifications, coding errors, and re-exports). The conceptual basis for using customs statistics is that, despite these limitations, they allow systematic and replicable international comparability, especially when using HS classifications and repositories such as. ^47^ Consequently, the framework does not equate reported trade with “sustainable trade”; instead, it treats reported trade as a partial but useful mirror of system functioning, whose interpretation is enriched by situating it within contemporary debates on governance, ^28^ traceability, ^15^ and risk. ^17^

Under this framework, the study is positioned as a comparative descriptive exercise: HHI represents the destination structure of HS 030743 by country and year, RSCA represents the country’s relative positioning in the global market for the same product, and both are reported and interpreted without introducing hypotheses about relationships between them, in line with a strictly descriptive scope oriented to sustainability audiences.

## 2. Materials and methods

The study was conducted under a quantitative approach with a strictly descriptive scope, based on secondary international trade statistics. Within this framework, the analysis characterized, for each country and year: (i) the diversification of export destination markets for HS 030743 (frozen cuttlefish and squid) using the Herfindahl–Hirschman Index (HHI), and (ii) the Revealed Symmetric Comparative Advantage (RSCA) as a bounded and centered measure of revealed specialization. Consistent with the descriptive objective, no associations, correlations, regressions, or any other procedures aimed at examining relationships between indicators were estimated; both analytical profiles were reported in parallel.

A non-experimental, retrospective longitudinal design was used, supported by annual export series by trading partner. The unit of analysis was the annual export flow (value) of product HS 030743 from each reporting country to each importing partner. Six exporting countries were included: China, Indonesia, India, Spain, Morocco, and Argentina. The observation period was delimited to 2020–2024, using only complete and comparable years; any year with partial or delayed information was excluded to avoid spurious distortions in the temporal description.

Data were obtained from UN Comtrade due to its global coverage and standardized reporter–partner–product (HS6)–flow structure, which supports replicability and international comparability. ^47^ Data extraction was performed through parameterized queries (reporter, partner, year, flow = exports, HS = 030743), and the query parameters and metadata were documented in accordance with the Comtrade+ methodological guide to ensure traceability of definitions, flows, and data treatments. ^48^

Data preparation prioritized internal consistency (rather than inferential “correction”), considering recent warnings about systematic issues in UN Comtrade (e.g., spatiotemporal statistical imbalances), which may bias descriptive results if basic cleaning criteria are ignored. ^49^ Accordingly, cleaning and harmonization routines were applied to standardize the year–reporter–partner–HS6–flow keys, following reproducible procedures for international trade data preparation. ^50^ As the main descriptive rule, exporter-reported values (FOB) were retained, without model-based reconciliation, in line with methodological reviews that recommend caution and transparency regarding outliers, missing data, and bilateral asymmetries in Comtrade. ^51^

Destination diversification was measured using the Herfindahl–Hirschman Index (HHI), computed from the distribution of HS 030743 export values across importing partners for each country and year. For country i , year t , and destination country j , the export share was defined as:

$$s_{\mathrm{ijt}}=\frac{X_{\mathrm{ijt}}}{\sum_{k\in J_{\mathrm{it}}}X_{\mathrm{ikt}}}$$

where $X_{\mathrm{ijt}}$ is the export value to destination j , and $J_{\mathrm{it}}$ denotes the set of all destination countries (i.e., all importing partners recorded in the database for country i in year t ) with positive exports of HS 030743. Thus, the HHI was calculated using the complete destination structure for each country-year, not a subset of partners. The index was then computed as:

$$\mathrm{HH}I_{\mathrm{it}}=\sum_{j\in J_{\mathrm{it}}}{\left(100\;s_{\mathrm{ijt}}\right)}^{2}.$$

On a 0–10,000 scale, where higher values indicate greater concentration and, therefore, lower diversification. For descriptive interpretation, conventional thresholds were used: HHI<1000 (unconcentrated destination markets; greater diversification), 1000≤HHI≤1800 (moderate concentration), and HHI>1800 (high concentration; lower diversification). ^52^ Nevertheless, HHI interpretation was handled with methodological caution, since the index may present interpretive validity limitations if destination granularity changes or if small shares are truncated; therefore, the analysis prioritized temporal trajectories and destination-structure context over mechanical threshold-based judgments. ^53^ ^,^ ^54^

Revealed specialization was assessed using the Revealed Symmetric Comparative Advantage (RSCA), which transforms the classical Balassa Revealed Comparative Advantage (RCA) into a symmetric and bounded scale. First, RCA was calculated for country i , product p (HS 030743), and year t as:

$$\mathrm{RC}A_{\mathrm{it}}=\frac{X_{\mathrm{ip},t}/X_{i\cdot ,t}}{X_{\cdot p,t}/X_{\cdot \cdot ,t}}$$

where $X_{\mathrm{ip},t}$ is country i ’s exports of HS 030743; $X_{i\cdot ,t}$ is country i ’s total exports (all products); $X_{\cdot p,t}$ is world exports of HS 030743; and $X_{\cdot \cdot ,t}$ is total world exports. The conceptual basis of RCA follows the foundational revealed comparative advantage approach based on export patterns. ^55^

Subsequently, the reported and analyzed indicator was its symmetric version:

$$\mathrm{RSC}A_{\mathrm{it}}=\frac{\mathrm{RC}A_{\mathrm{it}}-1}{\mathrm{RC}A_{\mathrm{it}}+1}$$

This indicator ranges from [−1,1] and is centered at 0 as the neutral point. This transformation was adopted because it reduces the inherent asymmetry of RCA and facilitates descriptive intertemporal and cross-country comparisons while preserving direct interpretation: positive values indicate relative revealed specialization, whereas negative values indicate relative non-specialization. This choice is supported by the literature introducing and applying RSCA in empirical studies, ^56^ as well as later reviews recognizing RSCA as a symmetric alternative for measuring specialization. ^57^

Finally, after data cleaning and harmonization, a country-year panel was constructed including: (i) total export value of HS 030743, (ii) the number of destination countries with positive exports, (iii) $\mathrm{HH}I_{\mathrm{it}}$ , and (iv) $\mathrm{RSC}A_{\mathrm{it}}$ . This panel provided the basis for the comparative description of diversification and specialization trajectories across the six exporting countries over the 2020–2024 period.

## 3. Results

### 3.1. China

Table 1 shows a clear pattern of commercial reconfiguration with progressive improvement in market diversification, even under export value volatility. The key finding is not only the decline in total exports in 2023 followed by a recovery in 2024, but also that concentration declines steadily (HHI from 1134 to 704). This is consistent with China moving from a moderately concentrated export structure to a more diversified one, compatible with a stronger ability to reallocate exports across destinations when some markets weaken.

**Table 1.** China’s squid and cuttlefish exports (USD millions), market diversification of China’s squid and cuttlefish exports, and RSCA of China’s squid and cuttlefish exports.

Importers	2020	2021	2022	2023	2024
Japan	211	215	266	230	220
United States of America	103	191	186	144	168
Spain	64	92	90	81	151
Korea, Republic of	269	206	296	176	181
Thailand	457	337	371	168	178
Others	781	1051	1106	760	799
Total	1885	2091	2314	1559	1697
**Years**	**2020**	**2021**	**2022**	**2023**	**2024**
HHI of export shares (0–10,000)	1134	949	850	771	704
**Importers**	**2020**	**2021**	**2022**	**2023**	**2024**
Japan	0.34	0.35	0.41	0.52	0.51
United States of America	−0.52	−0.31	−0.34	−0.23	−0.20
Spain	0.52	0.61	0.54	0.63	0.77
Korea, Republic of	0.53	0.38	0.48	0.44	0.45
Thailand	0.85	0.77	0.76	0.66	0.63

Source: UN Comtrade ^47^; authors’ calculations.

An important internal pattern is that the improvement in HHI occurs even while some traditional destinations lose relative weight, especially Thailand and, to a lesser extent, Korea. Analytically, this indicates that diversification is not driven only by “opening new markets,” but also by a reduction in dependence on historically dominant buyers. At the same time, the “Others” category remains large throughout the period, which reveals a broad destination base capable of cushioning shocks in specific markets.

From a competitiveness perspective, RSCA reflects an interesting repositioning: China maintains positive comparative advantages in Japan, Spain, Korea, and Thailand, but with different trajectories. The advantage in Spain strengthens markedly, and Japan also shows a stronger RSCA, compatible with improved positioning in demanding markets. In contrast, Thailand remains positive but declines over time, co-varying with stronger competition from other exporters and with a lower relative weight of Thailand in China’s export portfolio (causal interpretation is not advanced here). The United States is particularly relevant: RSCA remains negative throughout the period, but it becomes progressively less negative,which is consistent with a reduction in China’s comparative disadvantage in that market, even if it has not yet achieved a revealed advantage.

### 3.2. Indonesia

Table 2 reveals a pattern that differs from China: Indonesia grows strongly in export value, but becomes increasingly dependent on a small number of markets. Total exports increase steadily across the period, which confirms strong commercial dynamism; however, HHI remains very high in all years (well above 1800), indicating a highly concentrated export structure.

**Table 2.** Indonesia’s squid and cuttlefish exports (USD millions), market diversification of Indonesia’s squid and cuttlefish exports, and RSCA of Indonesia’s squid and cuttlefish exports.

Importers	2020	2021	2022	2023	2024
China	198	279	270	371	391
Viet Nam	92	95	184	142	224
Taipei, Chinese	74	55	51	57	46
Italy	10	12	24	14	16
Korea, Republic of	9	9	12	13	11
Others	42	43	46	30	39
Total	425	492	587	627	727
**Years**	**2020**	**2021**	**2022**	**2023**	**2024**
HHI of export shares (0–10,000)	2964	3735	3211	4114	3894
**Importers**	**2020**	**2021**	**2022**	**2023**	**2024**
China	0.41	0.42	0.34	0.40	0.39
Viet Nam	0.75	0.73	0.83	0.77	0.79
Taipei, Chinese	0.75	0.57	0.49	0.56	0.43
Italy	0.39	0.32	0.58	0.46	0.44
Korea, Republic of	−0.29	−0.36	−0.36	−0.31	−0.45

Source: UN Comtrade ^47^; authors’ calculations.

The main internal pattern is the consolidation of a China–Viet Nam axis as the core of Indonesia’s export growth. Over time, these two markets account for a rising share of total exports, while the “Others” category loses relative importance. This is consistent with Indonesia expanding successfully but with a less distributed commercial structure. Such a configuration is efficient in the short run and is associated with greater exposure if either of these key markets changes demand, trade conditions, or sanitary requirements.

The RSCA pattern reinforces this interpretation. Indonesia shows positive and relatively stable comparative advantages in China and especially in Viet Nam, confirming strong integration into the Asian trade circuit. However, Taipei, Chinese shows a gradual weakening in RSCA, suggesting a loss of relative specialization in that destination. Korea, Republic of remains negative and worsens over time, indicating a persistent and deepening comparative disadvantage. In other words, Indonesia is not broadening competitiveness uniformly; instead, it is deepening a geographically concentrated specialization.

### 3.3. India

Table 3 shows a more balanced export structure: India records growth until 2022 followed by an adjustment, while maintaining moderate and relatively stable concentration (HHI around 1260–1405). This is important because it suggests that the decline in total exports in 2023–2024 is not the result of excessive concentration, but more likely reflects demand or price effects, while the market structure remains comparatively stable.

**Table 3.** India’s squid and cuttlefish exports (USD millions), market diversification of India’s squid and cuttlefish exports, and RSCA of India’s squid and cuttlefish exports.

Importers	2020	2021	2022	2023	2024
Spain	125	154	201	148	161
Italy	60	102	117	107	102
Thailand	74	66	98	110	92
China	51	60	47	64	46
United States of America	20	46	61	42	36
Others	139	173	204	194	174
Total	468	600	727	665	610
**Years**	**2020**	**2021**	**2022**	**2023**	**2024**
HHI of export shares (0–10,000)	1371	1318	1405	1260	1389
**Importers**	**2020**	**2021**	**2022**	**2023**	**2024**
Spain	0.92	0.92	0.93	0.91	0.92
Italy	0.78	0.79	0.79	0.78	0.79
Thailand	0.84	0.77	0.82	0.87	0.86
China	0.22	0.26	0.32	0.44	0.37
United States of America	−0.62	−0.41	−0.36	−0.47	−0.51

Source: UN Comtrade ^47^; authors’ calculations.

The strongest pattern in the table is the presence of a stable “triad” of markets (Spain, Italy, and Thailand), which supports India’s export base without creating overwhelming dependence on a single destination. Unlike Indonesia or Morocco, India does not rely excessively on one buyer. In addition, the “Others” category remains consistently relevant, serving as a buffer. This reflects a functional diversification pattern: not highly fragmented, but sufficiently distributed to reduce vulnerability.

In competitiveness terms, India exhibits one of the most consistent profiles in the sample. RSCA is high and persistently positive in Spain, Italy, and Thailand, indicating strong and stable specialization in those destinations. The case of China is also notable because RSCA improves over the period (despite some fluctuation at the end), which suggests a stronger relative position there. By contrast, the United States remains negative throughout the period; although export values are not negligible, India’s relative competitive position in that market has not reached revealed comparative advantage.

### 3.4. Spain

Table 4 highlights a much larger export scale than the other countries, with a pattern of rapid expansion until 2022, followed by a moderate correction in 2023–2024. The key analytical point is that, despite this very large trade volume, concentration remains high, indicating substantial dependence on a few markets, especially China and the United States.

**Table 4.** Spain’s squid and cuttlefish exports (USD millions), market diversification of Spain’s squid and cuttlefish exports, and RSCA of Spain’s squid and cuttlefish exports.

Importers	2020	2021	2022	2023	2024
China	1853	2263	4247	3865	3234
United States of America	654	1182	1510	1364	1463
Spain	242	332	399	383	361
Italy	140	174	231	266	326
France	184	280	269	200	247
Others	554	859	1115	1013	1244
Total	3627	5090	7769	7093	6875
**Years**	**2020**	**2021**	**2022**	**2023**	**2024**
HHI of export shares (0–10,000)	3037	2619	3426	3402	2747
**Importers**	**2020**	**2021**	**2022**	**2023**	**2024**
China	0.53	0.52	0.51	0.50	0.52
United States of America	−0.14	−0.02	−0.18	−0.11	0.02
Spain	0.43	0.40	0.28	0.33	0.28
Italy	0.21	0.24	0.27	0.30	0.37
France	0.58	0.58	0.52	0.45	0.49

Source: UN Comtrade ^47^; authors’ calculations.

The central pattern is the dominance of the China–United States pair, which explains a large share of total exports throughout the period. When China’s absorption rises, HHI increases (as in 2022–2023); when the relative weight of other destinations grows (including “Others” and Italy), concentration declines somewhat (as in 2024). Therefore, HHI dynamics in Spain co-vary with fluctuations in Chinese demand. This is consistent with a large-scale export structure, but one that remains sensitive to the distribution of sales across a few major destinations.

The RSCA results provide a more nuanced reading: Spain maintains positive comparative advantages in China and France, and shows a clear improvement in Italy, compatible with stronger positioning within the European/Mediterranean trade network. The United States is especially noteworthy because RSCA shifts from negative or near-neutral levels to slightly positive in 2024,a pattern compatible with improved relative competitiveness or a more favorable export composition (without inferring causation). By contrast, the destination labeled Spain reflects reporter-partner self-flows in UN Comtrade, typically re-exports, re-imports, or coding mismatches. These rows are kept in the table for transparency but are not treated as external dependence; recomputing the indices without them does not change Spain’s concentration reading.

### 3.5. Morocco

Table 5 shows one of the most concentrated export structures in the entire analysis. Although Morocco records years of strong export growth, the dominant pattern is extreme dependence on Spain and Italy, with persistently very high HHI and peaks indicating severe concentration. Strategically, this is consistent with Morocco growing rapidly in certain years and with high vulnerability associated with limited destination diversification.

**Table 5.** Morocco’s squid and cuttlefish exports (USD millions), market diversification of Morocco’s squid and cuttlefish exports, and RSCA of Morocco’s squid and cuttlefish exports.

Importers	2020	2021	2022	2023	2024
Spain	177	255	238	310	272
Italy	95	124	53	113	126
Greece	4	3	3	6	6
Portugal	3	2	7	5	5
Japan	4	2	2	6	3
Others	29	19	11	28	5
Total	312	405	313	468	417
**Years**	**2020**	**2021**	**2022**	**2023**	**2024**
HHI of export shares (0–10,000)	4177	4904	6053	4980	5176
**Importers**	**2020**	**2021**	**2022**	**2023**	**2024**
Spain	0.41	0.49	0.59	0.49	0.49
Italy	0.75	0.76	0.58	0.64	0.71
Greece	0.68	0.56	0.54	0.62	0.66
Portugal	−0.22	−0.45	0.22	−0.19	−0.14
Japan	0.36	−0.10	−0.20	0.54	0.32

Source: UN Comtrade ^47^; authors’ calculations.

The most important internal pattern is that Spain functions as the anchor market, and changes in Spain’s purchases explain much of the variation in Morocco’s total exports. Italy acts as a second pillar, but with greater volatility. The “Others” category declines sharply toward the end of the period, implying an even less distributed market structure. This not only increases commercial risk but also reduces Morocco’s ability to offset downturns through alternative destinations.

In RSCA terms, Morocco maintains positive comparative advantages in Spain, Italy, and Greece, confirming strong competitiveness within the Mediterranean area. However, this competitiveness is geographically concentrated. Portugal shows an unstable pattern (mostly negative or near zero), and Japan shifts sign across the period, indicating a less consolidated position outside the core regional corridor. In other words, Morocco is competitive, but its competitiveness is embedded in a narrow regional network rather than spread across a broad global market base.

### 3.6. Argentina

Table 6 presents a very illustrative pattern: Argentina moves from an initially more concentrated structure (with strong dependence on China) to a more distributed configuration between 2021 and 2023, and then shows partial reconcentration in 2024 as the Chinese market recovers. This is clearly reflected in HHI: it falls sharply after 2020 (deconcentration) and rises again in 2024 (renewed concentration).

**Table 6.** Argentina’s squid and cuttlefish exports (USD millions), market diversification of Argentina’s squid and cuttlefish exports, and RSCA of Argentina’s squid and cuttlefish exports.

Importers	2020	2021	2022	2023	2024
China	195	60	56	63	118
Thailand	26	26	55	42	30
Spain	40	27	30	24	35
Japan	14	9	12	19	15
Korea, Republic of	51	61	72	68	58
Others	63	63	69	72	96
Total	389	247	293	288	352
**Years**	**2020**	**2021**	**2022**	**2023**	**2024**
HHI of export shares (0–10,000)	2875	1546	1573	1516	1783
**Importers**	**2020**	**2021**	**2022**	**2023**	**2024**
China	0.68	0.51	0.36	0.48	0.64
Thailand	0.80	0.90	0.96	0.95	0.92
Spain	0.67	0.65	0.68	0.59	0.69
Japan	0.73	0.61	0.64	0.74	0.84
Korea, Republic of	0.82	0.84	0.83	0.86	0.83

Source: UN Comtrade ^47^; authors’ calculations.

The key internal pattern is the change in the leading market: China dominates in 2020, then Korea becomes the main destination in 2021–2023, and China returns to a leading role in 2024. This shift coincides with export volatility and with a redistribution of flows across markets. In addition, the increasing weight of “Others” toward the end of the period suggests that Argentina expanded its destination base, although not enough to prevent China’s rebound from increasing concentration again.

From the competitiveness perspective, Argentina shows a very favorable feature: RSCA is consistently high and positive in all reported markets (China, Thailand, Spain, Japan, and Korea). This implies that Argentina’s main challenge is not product competitiveness, but rather instability in the geographic allocation of export flows or shifts in external demand. The case of China is especially revealing: RSCA declines and then recovers, mirroring the loss and return of China’s market leadership. At the same time, Korea and Thailand maintain very high RSCA values, confirming that Argentina preserves strong specialization in Asia even when the leading buyer changes.

As shown in Figure 1, China exhibits a more distributed portfolio in 2024, with a lower relative weight of the largest named destination and a broader combined participation of secondary destinations, which is consistent with a diversification pattern. Indonesia, in contrast, shows a stronger concentration in its top destinations by 2024, as the largest and second-largest named markets account for a greater share of total exports and the residual “Others” segment becomes less prominent. India maintains a comparatively stable portfolio composition across both years, reflecting a moderate and relatively balanced structure rather than sharp reconfiguration. Spain and Morocco remain highly concentrated, although with different profiles. Spain preserves a large dependence on its top destinations despite some redistribution among lower-ranked markets, while Morocco displays the most concentrated structure in the comparison, with an especially dominant top destination and a very limited residual share in 2024. Argentina presents an intermediate but dynamic pattern: the portfolio remains centered on a few leading destinations, yet the relative composition across ranked segments shifts between 2020 and 2024, suggesting a reallocation of export shares rather than a fully stable market structure. Overall, the figure provides a clear descriptive complement to the HHI results by visually summarizing how concentration/diversification is embedded in the ranked destination portfolio of each country.

**Figure 1.**
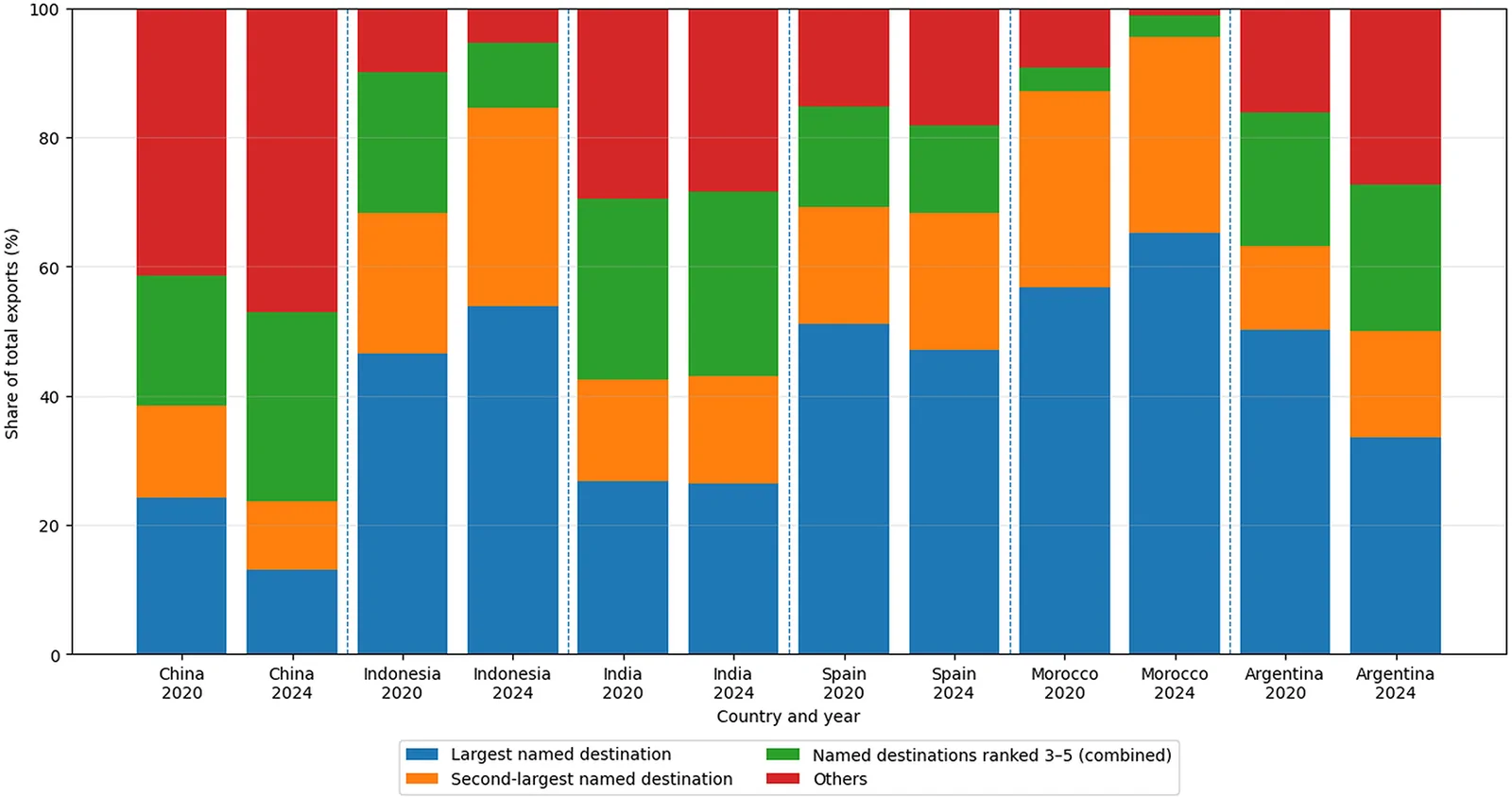
Comparative destination portfolio structure (Ranked shares), 2020 vs. 2024.

Figure 2 shows that India and Argentina display the most stable and favorable profiles, with consistently high and positive RSCA values across most reported destinations, indicating sustained relative export positioning over time. China also maintains positive RSCA values in key destinations (Japan, Spain, Korea, and Thailand), but with a clear internal reconfiguration: RSCA strengthens in Spain and remains solid in Japan, while it weakens in Thailand. In the United States, China’s RSCA remains negative in both years, although the shift toward zero suggests a reduction in relative disadvantage by 2024.

**Figure 2.**
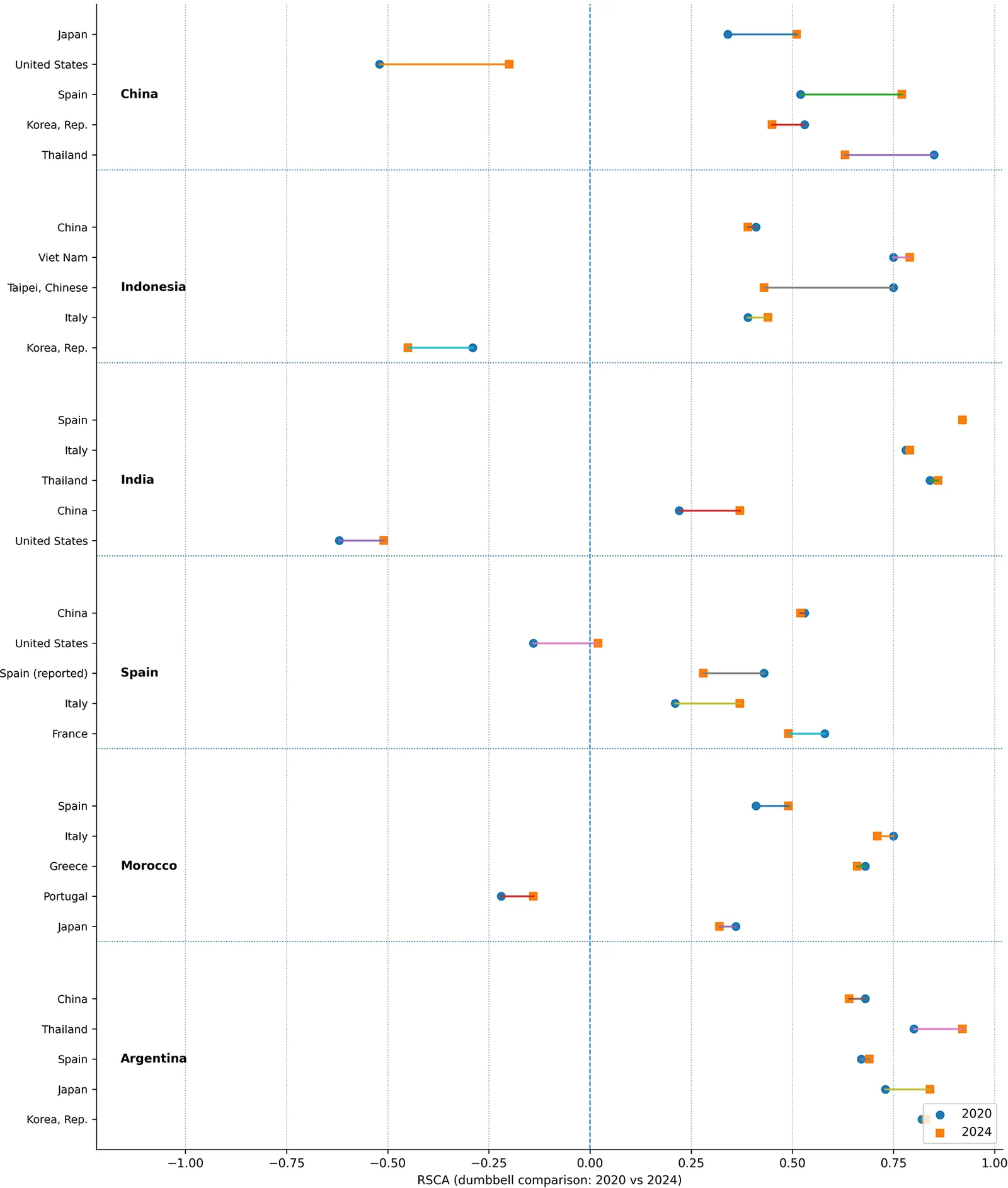
Changes in RSCA by destination market between 2020 and 2024 for HS 030743 exports: A Dumbbell plot.

The figure also highlights more selective or geographically concentrated specialization patterns. Indonesia retains positive RSCA in China, Viet Nam, Taipei (Chinese), and Italy, but the decline in Taipei and the persistently negative RSCA in Korea indicate that its competitive positioning is uneven across destinations. Spain presents a mixed profile, with positive RSCA in China, France, and Italy and a modest improvement in the United States (approaching neutrality in 2024), while destination-specific variation remains evident. Morocco maintains positive RSCA in its core Mediterranean destinations (Spain, Italy, and Greece), but with greater instability in Portugal and Japan. Overall, Figure 2 confirms that RSCA provides a complementary descriptive perspective to destination concentration (HHI), because it reveals where countries strengthen or weaken their relative positioning, not only how concentrated their export portfolios are.

In Figure 3, the most notable result is China’s strong deconcentration, with HHI declining from 1134 in 2020 to 704 in 2024, which implies a transition from a moderately concentrated structure to an unconcentrated destination portfolio. India remains comparatively stable within the moderate-concentration range (1371 to 1389), indicating a relatively balanced export structure with limited net change over the period. Argentina shows a substantial decline in concentration (2875 to 1783), moving from high concentration toward the upper bound of the moderate range, which is consistent with a more distributed destination structure by 2024 despite partial reconcentration relative to 2021–2023.

**Figure 3.**
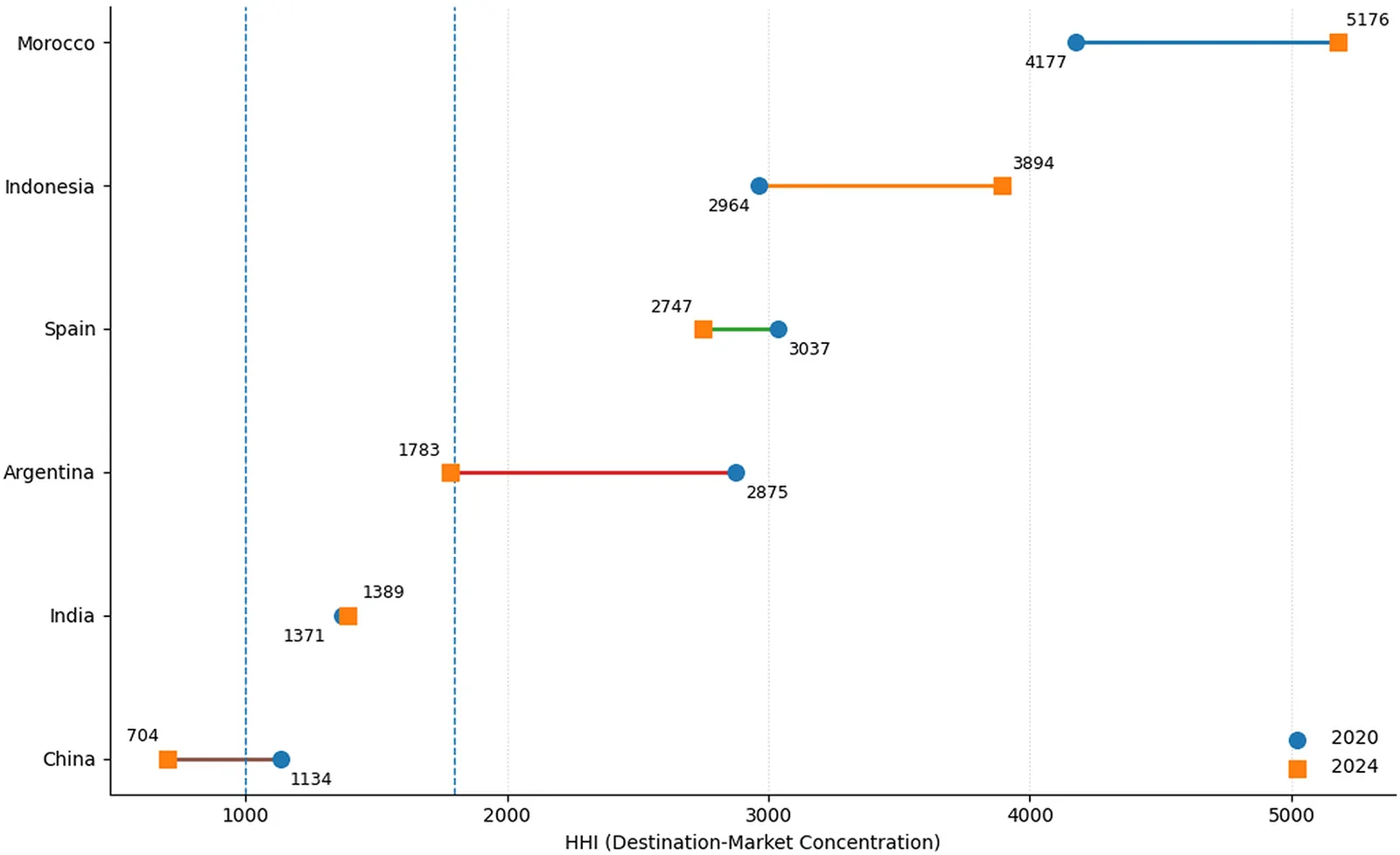
Changes in destination-market concentration (HHI) between 2020 and 2024 for HS 030743 Exports: A Dumbbell plot.

By contrast, Spain, Indonesia, and Morocco remain in the high-concentration zone in both years, although with different trajectories. Spain records a moderate reduction in HHI (3037 to 2747), suggesting some improvement in destination distribution, but concentration remains clearly high. Indonesia increases from 2964 to 3894, indicating a stronger dependence on a reduced set of destinations by 2024. Morocco also becomes more concentrated, rising from 4177 to 5176, and remains the most concentrated case in the sample. Overall, Figure 3 reinforces the descriptive interpretation that destination diversification dynamics differ markedly across countries: some cases improve through deconcentration (especially China and, to a lesser extent, Argentina and Spain), whereas others deepen concentration (especially Indonesia and Morocco). The figure therefore provides a clear comparative baseline for discussing commercial exposure and portfolio structure without inferring causal mechanisms.

## 4. Discussion

The results describe a heterogeneous export landscape for HS 030743, where differences in destination structure are as relevant as differences in export scale. China and India present comparatively more balanced configurations, but through distinct trajectories: China moves toward lower concentration over time, whereas India maintains a relatively stable, moderately concentrated portfolio. This distinction is important in descriptive terms because it shows that diversification can emerge either through gradual deconcentration or through sustained structural balance, without implying a common underlying mechanism. By contrast, Indonesia, Spain, and Morocco remain persistently concentrated, although around different commercial cores (Asian corridor, China–United States axis, and Mediterranean markets, respectively), which is consistent with the broader literature showing that cephalopod trade is globally extensive but still shaped by dominant nodes and asymmetric market linkages. ^30^ ^,^ ^33^ Argentina, in turn, displays a more volatile pattern, with a visible reallocation of flows across Asian destinations and only partial stabilization by the end of the period, reinforcing the idea that destination structure itself is a meaningful descriptive indicator of commercial exposure. ^41^

A second relevant finding is that the RSCA profiles add a distinct layer of interpretation that does not mechanically replicate HHI behavior. This supports the methodological decision of the study to treat diversification (HHI) and revealed specialization (RSCA) as parallel descriptive dimensions, rather than as variables linked by a causal hypothesis, in line with the literature on comparative advantage indicators and their symmetric transformation. ^9^ ^,^ ^56^ India and Argentina show the most stable positive RSCA patterns across their main destinations, suggesting strong revealed specialization even when their export values fluctuate. China, by contrast, shows a more dynamic repositioning process, with stronger relative performance in selected markets such as Spain and Japan, while gradually reducing its comparative disadvantage in the United States. Indonesia also exhibits positive RSCA in key destinations, but this competitiveness is concentrated in a narrow set of buyers, which means that strong specialization does not necessarily translate into broad market dispersion. Spain and Morocco similarly combine positive RSCA in core markets with concentrated destination structures, indicating that competitive strength can coexist with dependence on a limited number of export outlets.

The discussion of these patterns is especially relevant for sustainability-oriented interpretation, but the results should be read within the study’s strictly descriptive scope. As the literature on cephalopod supply chains emphasizes, trade performance alone does not capture governance quality, traceability integrity, or compliance conditions, all of which are central to sustainability assessments. ^15^ ^,^ ^16^ For this reason, the patterns observed in HHI and RSCA are interpreted here as descriptive signals of commercial resilience or vulnerability, not as evidence of sustainable or unsustainable fisheries outcomes. For example, lower concentration may indicate a more distributed destination portfolio and potentially lower dependence risk, but it does not by itself reveal whether the underlying supply chain is better governed. Likewise, strong RSCA values indicate relative export positioning, yet they may coexist with ecological variability or operational pressures that are common in squid and cuttlefish fisheries, where environmental fluctuations can affect supply conditions independently of market strategy. ^19^ ^,^ ^24^ This is precisely why the present analysis remains focused on characterization rather than causal attribution.

## 5. Conclusions

In conclusion, the study identifies three broad descriptive export configurations for frozen squid and cuttlefish: a comparatively diversified and more resilient profile (China, and in a stable form India), a growth-oriented but concentrated profile (Indonesia and Spain), and a specialized yet structurally vulnerable profile due to destination dependence (Morocco, with Argentina showing partial vulnerability through market reorientation volatility). This typology extends the trade-network and specialization literature by offering a replicable cross-country mapping of destination concentration and relative export positioning based on reported customs statistics. At the same time, and consistent with the study’s non-causal design, these findings should be understood as a baseline for further research rather than as explanatory evidence. Future work may build on this descriptive framework by integrating trade indicators with traceability, governance, and ecological variables to produce a more comprehensive sustainability assessment of cephalopod export systems.

Based on the descriptive evidence, the recommended action is to tailor trade monitoring and export strategy to each country’s destination structure rather than applying a single policy logic to all cases. Countries with broader or improving diversification (especially China, and in a more stable form India) should prioritize preserving destination breadth while strengthening traceability and compliance across markets, because diversification improves commercial resilience but does not, by itself, demonstrate sustainability performance. Countries with persistent concentration despite export growth (especially Indonesia and Spain) should gradually expand their destination portfolio to reduce dependence on a small number of anchor buyers, while countries with strong specialization but narrow destination dependence (especially Morocco, and partially Argentina) should prioritize risk management by maintaining core markets and developing secondary outlets. In all cases, HHI and RSCA should be monitored jointly as complementary descriptive indicators of market structure and relative export positioning, without treating either indicator as evidence of causality or as a substitute for governance and sustainability assessment.

Given the study’s strictly descriptive and non-causal design, future research should treat these results as a baseline for extension rather than as evidence of determinants. The main recommendation is to integrate customs-based indicators (HHI and RSCA) with variables that capture dimensions absent from trade records (especially traceability, governance quality, labor conditions, and ecological variability) so that sustainability can be assessed more comprehensively. In methodological terms, future studies should also strengthen robustness by using mirror statistics, sensitivity checks for destination granularity, longer time series, and (where possible) greater product disaggregation beyond HS 030743 to distinguish species and processing profiles. Network-based trade indicators may also be added to refine structural interpretation, but they should be used under the same caution maintained in this study: they enhance descriptive mapping and comparative diagnosis, but they do not establish causal relationships.

## Institutional review board statement

Not applicable.

## Informed consent statement

Not applicable.

## Data Availability

Figshare: Mapping export sustainability in frozen cephalopods: destination concentration and revealed comparative advantage in China, Indonesia, India, Spain, Morocco, and Argentina (2020–2024). https://doi.org/10.6084/m9.figshare.32441517 ^58^ This project contains the following underlying data:
- Country–year export records for HS 030743 (frozen cuttlefish and squid) covering 2020–2024, organized by reporter country, importing partner, year, Harmonized System code, and export value (free on board, in United States dollars), derived from the United Nations International Trade Statistics Database (UN Comtrade). - Computed Herfindahl–Hirschman Index (HHI) values per country and year, including the destination shares used in their calculation. - Computed Revealed Symmetric Comparative Advantage (RSCA) values per country, destination market, and year. - Underlying values for all reported tables ( Tables 1– 6) and figures ( Figures 1– 3) of the manuscript. No human-subject data are included. Data are available under the terms of the Creative Commons Attribution 4.0 International license (CC-BY 4.0).
