# Mapping Export Sustainability in Frozen Cephalopods: Destination Concentration and Revealed Comparative Advantage in China, Indonesia, India, Spain, Morocco, and Argentina (2020–2024)

**DOI:** 10.12688/f1000research.183651.2

**Published:** 2026-10-08

**Authors:** Jose Carlos Montes Ninaquispe, Luisa Angelica Orejuela Guerrero, Eleodora del Pilar Orejuela Guerrero, Carlos José Sandoval Reyes, José Alfredo Castañeda Nassi, Victor Ángel Ancajima Miñán, Rosario Yaqueliny Llauce Santamaria, Juan Carlos Damián Sandoval, Karina Tomasa Valdiviezo Pérez, César Pol Arévalo-Aranda, Marco Agustín Arbulú Ballesteros

**Affiliations:** 1 Programa de Administración y Administración de Negocios Internacionales Filial Norte, Universidad de San Martín de Porres, Chiclayo, 14001, Peru; 2 Departamento de Ingeniería, Escuela Profesional de Ingeniería Industrial, Facultad de Ingeniería, Universidad Nacional de Trujillo, Trujillo, La Libertad, 13011, Peru; 3 Departamento de Ingeniería, Escuela Profesional de Ingeniería Ambiental, Facultad de Ingeniería, Universidad Privada del Norte, Campus San Isidro-Trujillo, Trujillo, La Libertad, 13001, Peru; 4 Escuela de Administración, Facultad de Ciencias Económicas, Universidad Privada Antenor Orrego, Trujillo, La Libertad, 13008, Peru; 5 Institute for Research in Science and Technology, César Vallejo University, Campus Chepén-Piura, Trujillo, La Libertad, 13001, Peru; 6 Departamento Académico de Ciencias Básicas y Aplicadas, Facultad de Ingeniería, Universidad Nacional de Jaén, Jaén, Cajamarca, 06801, Peru; 7 Departamento Académico de Ingeniería Metalúrgica, Escuela Profesional de Ingeniería Metalúrgica, Universidad Nacional de Trujillo, Trujillo, La Libertad, 13011, Peru

**Keywords:** export diversification; revealed comparative advantage; Herfindahl–Hirschman Index; frozen cephalopods; HS 030743; UN Comtrade; seafood trade; export concentration.

## Abstract

**Background:**

Frozen squid and cuttlefish (Harmonized System code 030743) form a commercially relevant segment of global cephalopod trade, with implications for cold-chain logistics, traceability and the economic resilience of exporting countries. Despite this relevance, comparable cross-country evidence on how export portfolios are structured and how relative specialization evolves remains limited. The study describes and compares destination diversification and revealed export positioning of frozen cephalopod exports for China, Indonesia, India, Spain, Morocco and Argentina between 2020 and 2024.

**Methods:**

A quantitative, non-experimental, retrospective longitudinal design was applied using secondary statistics from the United Nations International Trade Statistics Database (UN Comtrade). Destination diversification was measured through the Herfindahl–Hirschman Index (HHI), computed from the complete distribution of export shares across importing partners. Revealed specialization was assessed through the Revealed Symmetric Comparative Advantage (RSCA), obtained from the symmetric transformation of the Balassa index. The description was complemented with summary statistics (means, standard deviations, coefficients of variation and concentration ratios) and with exploratory non-parametric tests (Kruskal–Wallis, Friedman and Spearman correlations) that compare countries and years without inferring causal mechanisms; cleaning and harmonization followed documented routines to ensure replicability.

**Results:**

Cross-country heterogeneity was marked. China showed sustained deconcentration, with HHI declining from 1,134 to 704. India maintained a stable, moderately concentrated portfolio. Argentina moved from high concentration toward a more distributed structure, with partial reconcentration in 2024. Indonesia deepened its concentration, with HHI rising from 2,964 to 3,894, and Morocco remained highly concentrated throughout. Spain preserved high concentration despite redistribution among secondary markets. Between-country differences in HHI were large (Kruskal–Wallis H = 27.4, p < 0.001, ε ^2^ = 0.95), whereas no common year effect was detected (Friedman χ ^2^ = 3.07, p = 0.547); across country–years, destination-level RSCA was moderately and inversely associated with concentration (Spearman ρ = −0.40, p = 0.030). RSCA values evidenced persistent revealed specialization for India and Argentina, China’s repositioning in selected destinations, Indonesia’s Asia-centered specialization, and Morocco’s competitiveness embedded in Mediterranean outlets.

**Conclusions:**

Three descriptive export profiles were identified: diversified and stable; growth-oriented yet dependent; and specialized but destination-vulnerable. Supported by explicit summary statistics and exploratory tests, this typology offers a replicable baseline for sustainability discussions without inferring causal mechanisms.

## 1. Introduction

Global exports of fisheries and aquaculture products have become strategically central to contemporary sustainability debates, not only because of their contribution to food security, but also because of their role in generating income, employment, and cross-border production linkages in increasingly globalized food systems. ^1^ In this context, cephalopods—particularly squid and cuttlefish—have consolidated their position as a resource group of high commercial and cultural relevance, with capture, processing, and trade dynamics that combine biological complexity (short life cycles and high interannual variability) with strong market pressures, regulatory demands, and traceability challenges. Trade metrics therefore matter beyond their economic meaning: they also act as indirect signals of vulnerability, dependence, and resilience in production systems exposed to environmental, regulatory, and demand shocks.

Within the broader universe of cephalopod trade, this study focuses on product HS 030743 (frozen cuttlefish and squid), as this category captures two features that are crucial for the sustainability of modern fisheries chains: (i) the predominance of a frozen format, which typically implies cold-chain infrastructure, international logistics, standardization, and, in many cases, industrial processing; and (ii) the possibility of observing, through customs data, relatively comparable cross-country patterns in terms of destinations, relative shares, and positioning in the global market. In line with standards for replicable research in trade economics and sustainability, the analysis relies on international trade statistics and harmonized classifications, which are widely used in applied seafood research. ^2^

The empirical focus is on six countries—China, Indonesia, India, Spain, Morocco, and Argentina—selected because of their recurrent presence in academic and policy discussions related to cephalopod production, fishing fleets, processing capacity, trade, and governance. China occupies a prominent place in debates on ocean fisheries and fleets operating beyond national jurisdictions, particularly squid fisheries associated with light-attraction vessels, which has motivated studies highlighting regulatory gaps and monitoring challenges. ^3^ In the Southwest Atlantic, Argentina is an emblematic case due to the interaction between exclusive economic zones, high-seas activity, and squid-related geopolitical tensions, with direct implications for sustainability and governance. ^4^ Spain, in turn, is embedded in the European seafood consumption and import system, where demand, regulation, and market standards jointly shape incentives for traceability and compliance. ^5^ Morocco is linked to debates on the political and economic configuration of fisheries sectors along the Northwest African Atlantic coast and their export projection toward demanding markets, in contexts where sustainability is intertwined with institutional disputes and arrangements. ^6^ Finally, India and Indonesia are associated with supply-side dynamics involving extraction and processing capacity, fleet heterogeneity, and governance and control challenges in complex marine product chains, so that a careful description of their export positioning is a necessary first step before any causal claim can be examined. ^7^ ^,^ ^8^

In terms of relevance, a diagnosis of destination diversification can help interpret exposure to risks arising from trade dependence, while the Revealed Symmetric Comparative Advantage (RSCA) allows a comparative assessment of the product’s relative export positioning against the global specialization pattern. ^9^ The contribution of this study, therefore, does not rest on causal inference, but on constructing an empirical and comparable “mapping” of how destinations are distributed and how each country’s relative positioning is expressed in the global market, as emphasized in studies that analyze these indicators. ^10^ ^–^ ^12^

This need for mapping becomes more pressing when one considers that the sustainability of seafood supply chains faces growing demands for transparency, traceability, and compliance, driven by both regulation and reputational and market pressures. ^13^ ^,^ ^14^ The literature on cephalopod traceability has warned that, compared with other groups (e.g., fish), monitoring and standardization mechanisms for cephalopods have developed unevenly, leaving persistent gaps in identification, documentation, and control along the chain. ^15^ Recent analyses also stress that “declared” traceability is not always equivalent to operational and verifiable traceability, and that current tools may be insufficient to sustain claims of sustainability and legality in global seafood chains. ^16^ In parallel, studies combining satellite data and empirical evidence have documented the coexistence of risks related to illegal, unreported, and unregulated fishing and labor abuses in certain fleet segments and regions, challenging any simplified sustainability narrative based solely on aggregate trade data. ^17^

In this context, international trade reflects not only market preferences or cost advantages, but also—often with lags and distortions—the outcomes of regulatory frameworks, control capacities, infrastructure, integration into global value chains, and quality standards. ^18^ This is particularly visible in squid fisheries: research on the underregulated nature of global squid fisheries has shown that, in the absence of robust governance arrangements, exploitation and supply dynamics can expand rapidly, driven by demand and technological capabilities for detection and capture. ^19^ At the macro-institutional level, attempts to realign incentives through trade and environmental disciplines (e.g., the Agreement on Fisheries Subsidies) further reinforce the importance of understanding how export specializations in marine products are configured and reconfigured. ^20^

Three reasons justify the study: the commercial centrality of frozen cephalopods, the presence of six countries with distinct profiles in the international trade of this product, and the need for comparable descriptive diagnoses that can inform sustainability discussions without forcing causal relationships. Accordingly, the general objective is to describe and compare, for China, Indonesia, India, Spain, Morocco, and Argentina, (1) destination diversification (HHI) and (2) the symmetric RCA (RSCA) of HS 030743 exports using a replicable international trade framework. Summary statistics and exploratory non-parametric comparisons are reported to support this description, while the design remains non-causal. The guiding research question is: how are destination concentration/diversification and relative export positioning for HS 030743 characterized and how do they evolve, comparatively, across the six selected countries within the global market?

### 1.1 Literature review

Recent cephalopod literature converges on the idea that, while their commercial importance has increased, major structural sustainability challenges persist in relation to biology, exploitation, and governance. A key contribution has been the development of integrated assessments combining pressure, state, and institutional response to estimate the sustainability of cephalopod fisheries across multiple countries, highlighting important heterogeneity among economies and showing that response capacities (governance, control, and stressor mitigation) are decisive for limiting risk. ^21^ These differences matter for trade analysis because supply stability, compliance costs, and market acceptance may vary across countries and translate into shifts in destinations, relative prices, or specialization.

A second line of research emphasizes that cephalopod system sustainability cannot be addressed without considering biological uncertainty and methodological limits in stock assessment. Because many cephalopod fisheries operate under data-limited conditions, with high environmental variability and short life cycles, “rapid” assessment approaches and alternative methods have been proposed to support more timely decisions, underscoring that limited high-frequency data infrastructure is itself a sustainability problem. ^22^ ^,^ ^23^ For trade analysis, this suggests that abrupt changes in exportable volumes may coexist with apparent stability in annual aggregates, reinforcing the need for descriptive approaches that do not confuse ecological variation with “trade strategy.”

Evidence has also strengthened the link between cephalopods and climate change, albeit in complex and non-uniform ways. A study on climate change impacts on cephalopods reported consistent signs of ecological sensitivity and biogeographic responses, showing that variations in temperature and oceanographic conditions can alter distribution, abundance, and catch conditions at regional scales. ^24^ This line of research does not replace trade analysis, but it helps contextualize why export series may exhibit episodes of expansion or contraction that, descriptively, appear as changes in destination shares or relative participation without requiring immediate causal interpretation.

Fishing-related criminality has also been examined through global approaches that reveal concentration patterns in fisheries crimes and the ability to identify actors and structures, reinforcing the idea that sustainability requires monitoring and enforcement instruments beyond production and trade metrics. ^25^ Although the present study does not estimate effects, it suggests that changes in destinations or specialization may occur in contexts where governance and control are under strain, particularly in high-seas fisheries and distant-water fleets.

In the specific case of squid, the literature has advanced by documenting the magnitude of regulatory gaps in global fisheries associated with light-attraction fleets and transboundary operations. ^19^ One study argued that a substantial share of global activity takes place under weak or nonexistent regulation, raising major sustainability and international governance dilemmas. ^19^ This diagnosis is particularly relevant for countries such as China and, by extension, adjacent regions, where high-seas fishing pressure has become a recurring topic in public and academic debate. ^26^

At the regional and geopolitical scale, the evidence shows that high-seas squid fishing—especially near national jurisdictions—can generate tensions at the intersection of sustainability and sovereignty. ^27^ An analysis focused on Chinese fleets operating near South American EEZs described squid fishing as a phenomenon that combines resource pressure, governance gaps, and geopolitical frictions. ^4^ Such studies help explain why Argentina, for example, occupies a distinctive place in squid-related debates and why trade in squid-derived products may be shaped by narratives of sustainability, surveillance, and control.

Complementary research has examined opportunities and gaps in international law for addressing the underregulation of high-seas squid fisheries. A recent legal review identified partial progress alongside persistent normative gaps and proposed opportunities for more effective management. ^28^ This discussion is relevant for trade because regulatory evolution may eventually translate into new requirements for documentation, traceability, and compliance, thereby reshaping export destinations and processing incentives.

Alongside governance, another important field concerns product traceability and authenticity. Evidence suggests that cephalopod markets may be exposed to species substitution and mislabeling problems, especially in processed products or those with morphologies that are difficult to distinguish. ^29^ A recent study applied to cephalopod markets documented mislabeling rates and discussed implications for fraud control, food safety, and market governance. In frozen products, where processing may reduce morphological cues, traceability becomes even more critical.

Consistently, the literature has warned that cephalopod traceability lags behind that of other seafood groups and that chain-wide standardization (from capture to commercialization) remains uneven. ^30^ A synthesis study emphasized that progress toward sustainability requires better integration of species identification, origin documentation, stock monitoring, and traceability across global cephalopod chains. ^15^ This body of work reinforces the point that trade, even when legal and reported, may not by itself capture critical sustainability attributes unless supported by verification systems.

Another set of studies has examined the role of states in promoting traceability and sustainability in fisheries, showing that policy design may produce non-trivial effects, including the displacement of problems and the reconfiguration of incentives along the chain. A recent study on state-led traceability discussed mechanisms through which traceability may support sustainability, but also highlighted risks of “externalization” when formal compliance is emphasized without addressing structural asymmetries. ^31^ This is particularly relevant because high-standard markets (such as several European markets) may induce changes in export destinations and trade forms, but such changes should not be interpreted automatically as substantive improvements without additional evidence.

Regarding social sustainability, the literature has also stressed that labor justice analyses in seafood supply chains can become constrained by methodological nationalism, which underestimates the transnational structure of these chains and complicates attribution of responsibility and remedy. A recent contribution showed how labor justice analysis benefits from a more explicitly transnational perspective in export-oriented marine product chains. ^32^ This perspective again reinforces why a descriptive trade diagnosis should be understood as one component of a broader sustainability puzzle.

In the literature specifically focused on international cephalopod trade, important advances have been made in mapping global trade networks using reported trade data. An influential study provided a long-run overview of legal cephalopod trade, identifying dominant actors, centrality patterns, and market asymmetries. ^30^ This type of evidence supports the present study’s approach by showing that, even without causal modeling, descriptive analysis of customs data can reveal structures of dependence and concentration across countries and destinations.

Another study on cephalopod markets highlighted that the global trade network includes a very large number of countries, but is dominated by a relatively small group of economies—particularly in Asia—with interactions among demand, food security, control systems, and authenticity. ^33^ This supports the selection of focal countries such as China and India and strengthens the argument that destination diversification and relative specialization may vary substantially according to industrial structures and historical trade linkages.

Within the literature on trade networks and fisheries sustainability, recent studies have used network metrics to examine global patterns, emphasizing that trade configuration may be associated with different sustainability scenarios and relative biomass conditions, thereby informing debates on how the architecture of international trade may support—or strain—sustainability objectives. ^34^ Although the present study does not aim to test relationships, this literature justifies why describing concentration/diversification and export positioning is useful for sustainability audiences: these are indicators of trade architecture that may amplify or buffer risks.

In resource-specific studies, there has also been growing interest in the spatial dynamics and abundance of Argentine squid (Illex argentinus) in the South Atlantic, with forecasting methods integrating oceanographic variables. A recent study on spatiotemporal abundance in the Falkland/Malvinas area improved understanding of resource availability in a key squid-producing zone, which is relevant for interpreting supply fluctuations that may be reflected in frozen-product exports. ^35^ This evidence supports the idea that, even in purely trade-based analyses, ecological variability is an indispensable interpretive context.

Meanwhile, studies on artisanal cephalopod fisheries in Northwest Africa have documented sustainability tensions in settings where the resource is socially and economically crucial. A Marine Policy study on the artisanal octopus fishery in Mauritania reported signs of decline and discussed implications for management, employment, and income, providing a regional framework that resonates with sustainability concerns in nearby Atlantic cephalopod systems. ^36^ This suggests that trade flows in frozen cephalopods may coexist with local sustainability pressures in exporting regions.

In Asia, where a substantial share of global cephalopod activity is concentrated, the literature has examined ecological and operational dimensions of squid-oriented fisheries. A study on purpleback flying squid (Sthenoteuthis oualaniensis) in the northwestern Indian Ocean used nonlinear models and machine learning to explain distribution and catches, highlighting the relevance of temporal and thermal factors in fishery performance. ^37^ Although biological-operational in focus, this work helps contextualize supply dynamics for China and associated fishing areas.

Similarly, other studies have analyzed squid fishing zones and fleets in the Indian Ocean with implications for distant-water fisheries. A recent study investigated factors influencing the location of squid jigging activity in the northwestern Indian Ocean, offering empirical evidence on spatial patterns of fishing effort and catches. ^38^ By emphasizing spatial and temporal heterogeneity, this literature reinforces interpretive caution: changes in trade metrics may reflect both resource conditions and market or logistical reconfiguration.

In Europe, the literature on marine sustainability and biodiversity has incorporated cephalopods into discussions of ecosystem-based management and environmental policy frameworks. A Marine Biology study examined the role of cephalopods under the EU Marine Strategy Framework Directive, providing evidence on monitoring and conservation challenges with indirect implications for European countries linked to cephalopod capture and consumption. ^39^ For a country such as Spain, embedded in the European seafood market, these debates form part of the regulatory and sustainability environment in which import and export flows operate.

Complementarily, conservation literature has stressed that cephalopod biodiversity management is hindered by knowledge and monitoring gaps, which may bias status assessment and management policies. A recent synthesis identified knowledge gaps and challenges for cephalopod conservation and management in contexts where exploitation is economically important. ^40^ This evidence helps explain why cephalopod sustainability requires integrated approaches that go beyond purely trade-based indicators.

From a methodological standpoint, the trade-and-sustainability literature recommends caution when interpreting foreign trade indicators as direct proxies for sustainability. The reason is twofold: reported trade data do not fully capture informality, underreporting, or externalities, and marine product chains may undergo reconfiguration driven by standards, certifications, and policy changes. A recent review on export diversification and economic vulnerability in African monetary unions showed, from an applied perspective, that concentration/diversification is associated with exposure to external shocks, reinforcing the usefulness of concentration as a descriptive risk indicator. ^41^ Although the object of that study is macroeconomic, the argument is transferable: an export portfolio concentrated in a few destinations can amplify vulnerability in product chains.

Finally, to complete the relevant literature landscape, methodological contributions on specialization indicators and revealed comparative advantage are essential, since the symmetric RCA (RSCA) is widely used. One study discussed how the RCA distribution tends to be non-normal and reviewed transformations and alternative indices, which is relevant for justifying why even descriptive studies should explicitly state the “symmetric” nature of the indicator and its interpretation. ^9^ In addition, recent studies have assessed RCA across sectors and economies, showing its usefulness for describing changes in relative specialization over time. ^42^ This literature supports the use of symmetric RCA as a descriptive tool, especially when the objective is cross-country comparability without extrapolating causal conclusions.

In summary, the literature review supports five ideas that justify the study: (i) cephalopod sustainability is heterogeneous and involves integrated challenges related to ecological pressure, governance, and institutional response; (ii) squid fisheries in particular face major regulatory gaps on the high seas, with sustainability and geopolitical implications; (iii) cephalopod traceability and market authenticity remain areas with persistent gaps, especially relevant for frozen products ^29^; (iv) reported trade patterns can be mapped using network approaches and provide useful evidence for describing structures of dominance and dependence; and (v) concentration/diversification and relative specialization metrics, even without causal inference, are appropriate descriptive tools for discussing risk, resilience, and sustainability in global chains.

### 1.2 Theoretical framework

The theoretical framework of this study integrates three components: (i) the conceptualization of international seafood trade as part of socioecological systems and global value chains; (ii) destination diversification/concentration as a descriptive attribute of the export “portfolio”; and (iii) relative specialization measured through symmetric RCA (RSCA) as an indicator of export positioning in the global market. These components are articulated without postulating causal relationships between the two central indicators (HHI and RSCA), because the scope is strictly descriptive.

First, international trade in fishery products can be understood as an observable manifestation of socioeconomic networks linking extraction, processing, logistics, regulation, and consumption. In cephalopods, this network is characterized by broad geographic coverage and the dominance of certain nodes, with clear implications for sustainability and governance. ^33^ From this perspective, customs data make it possible to observe flows, destinations, and relative shares, although they do not by themselves reveal internal sustainability attributes (e.g., legality, stock status, or decent work conditions). Accordingly, the framework adopted here treats trade indicators as descriptive “layers” that must be interpreted within a broader sustainability perspective in which traceability and governance are enabling conditions for aligning trade flows with environmental and social objectives. ^16^

Second, destination diversification is theoretically grounded in the portfolio concept: an exporting country distributes its supply across multiple destination markets, and that distribution may be more concentrated or more diversified. ^43^ The Herfindahl–Hirschman Index (HHI) is widely used to capture this structure through the sum of squared destination shares within the product’s total exports. Conceptually, higher values indicate concentration (dependence on a few destinations), whereas lower values indicate a more balanced distribution across markets. From the standpoint of economic sustainability, this structure is linked to exposure to external shocks, regulatory changes, demand variation, or logistics disruptions, making it a useful descriptive risk diagnostic. ^41^ For a product such as HS 030743, where cold-chain logistics and sanitary compliance can condition market access, destination diversification may be interpreted as an attribute of commercial resilience, without inferring that such resilience derives from any specific cause.

In applied sustainability and trade studies, diversification and competitiveness have been proposed as complementary dimensions for discussing export sustainability, although empirical evidence and mechanisms vary by sector. A study published in *Sustainability* discusses export “sustainability” through diversification and competitiveness in comparative analyses, illustrating how these metrics can be used for characterization and diagnosis without assuming invariant relationships between indicators. ^44^ In the present study, that perspective is adopted only in its descriptive dimension: HHI is interpreted as the destination structure of HS 030743 by country and year.

Third, symmetric Revealed Comparative Advantage (RSCA) is grounded in the tradition of international specialization “revealed” through export patterns. In its classical form, RCA compares the share of a product in a country’s exports with the share of the same product in world exports. It is considered “asymmetric” because its scale is not centered or bounded symmetrically: values below 1 indicate revealed comparative disadvantage, while values above 1 indicate revealed comparative advantage, but the magnitude above 1 can increase without an upper bound equivalent to the lower bound. ^9^ This asymmetry has motivated extensive discussion on statistical properties, comparability, and alternative transformations, yet RCA remains attractive because of its simplicity and interpretability as an indicator of relative specialization.

Even so, in descriptive trade studies—especially in agri-food and natural resource products—RCA is typically used to trace specialization profiles by country and their temporal evolution, rather than to explain underlying causes. ^45^ Consistent with this use, the present study adopts symmetric RCA (RSCA) as an indicator of the relative positioning of HS 030743, reported by country and year, without using it as an explanatory variable for any other indicator.

Another relevant strand of literature examines the link between comparative advantage measured in gross exports and production fragmentation in global value chains, which may alter the interpretation of specialization when an economy participates in specific processing stages or re-export activities. A recent study, from a value-added trade perspective, proposed that comparative advantage can be analyzed using dynamic methodologies, emphasizing that interpretations change when value-added content is considered. ^46^ This discussion is pertinent for HS 030743 because frozen products may involve different degrees of processing and international linkages; therefore, the theoretical framework recognizes interpretive limits of RCA/RSCA in the presence of productive fragmentation, while the study itself remains at the descriptive level of recorded exports.

Finally, because the empirical object is HS 030743 exports, the framework assumes the validity of the “reported trade” approach as an approximation to recorded legal flows, while recognizing known limitations (underreporting, reclassifications, coding errors, and re-exports). The conceptual basis for using customs statistics is that, despite these limitations, they allow systematic and replicable international comparability, especially when using HS classifications and repositories such as UN Comtrade. ^47^ Consequently, the framework does not equate reported trade with “sustainable trade”; instead, it treats reported trade as a partial but useful mirror of system functioning, whose interpretation is enriched by situating it within contemporary debates on governance, ^28^ traceability, ^15^ and risk. ^17^

Under this framework, the study is positioned as a comparative descriptive exercise: HHI represents the destination structure of HS 030743 by country and year, RSCA represents the country’s relative positioning in the global market for the same product, and both are reported and interpreted as parallel descriptive layers; the exploratory associations examined between them are screening tools, not hypotheses about mechanisms, in line with a descriptive scope oriented to sustainability audiences.

## 2. Materials and methods

The study was conducted under a quantitative approach with a strictly descriptive scope, based on secondary international trade statistics. Within this framework, the analysis characterized, for each country and year: (i) the diversification of export destination markets for HS 030743 (frozen cuttlefish and squid) using the Herfindahl–Hirschman Index (HHI), and (ii) the Revealed Symmetric Comparative Advantage (RSCA) as a bounded and centered measure of revealed specialization. Consistent with the descriptive objective, both analytical profiles were reported in parallel and complemented with summary statistics and exploratory non-parametric comparisons (Section 2.1) that screen for differences among countries and years and for monotonic associations, without modelling causal relationships between the indicators.

A non-experimental, retrospective longitudinal design was used, supported by annual export series by trading partner. The unit of analysis was the annual export flow (value) of product HS 030743 from each reporting country to each importing partner. Six exporting countries were included: China, Indonesia, India, Spain, Morocco, and Argentina. The observation period was delimited to 2020–2024, using only complete and comparable years; any year with partial or delayed information was excluded to avoid spurious distortions in the temporal description.

Data were obtained from UN Comtrade due to its global coverage and standardized reporter–partner–product (HS6)–flow structure, which supports replicability and international comparability. ^47^ Data extraction was performed through parameterized queries (reporter, partner, year, flow = exports, HS = 030743), and the query parameters and metadata were documented in accordance with the Comtrade+ methodological guide to ensure traceability of definitions, flows, and data treatments. ^48^

Data preparation prioritized internal consistency (rather than inferential “correction”), considering recent warnings about systematic issues in UN Comtrade (e.g., spatiotemporal statistical imbalances), which may bias descriptive results if basic cleaning criteria are ignored. ^49^ Accordingly, cleaning and harmonization routines were applied to standardize the year–reporter–partner–HS6–flow keys, following reproducible procedures for international trade data preparation. ^50^ As the main descriptive rule, exporter-reported values (FOB) were retained, without model-based reconciliation, in line with methodological reviews that recommend caution and transparency regarding outliers, missing data, and bilateral asymmetries in Comtrade. ^51^

Destination diversification was measured using the Herfindahl–Hirschman Index (HHI), computed from the distribution of HS 030743 export values across importing partners for each country and year. For country i , year t , and destination country j , the export share was defined as:

$$s_{\mathrm{ijt}}=\frac{X_{\mathrm{ijt}}}{\sum_{k\in J_{\mathrm{it}}}X_{\mathrm{ikt}}}$$

where $X_{\mathrm{ijt}}$ is the export value to destination j , and $J_{\mathrm{it}}$ denotes the set of all destination countries (i.e., all importing partners recorded in the database for country i in year t ) with positive exports of HS 030743. Thus, the HHI was calculated using the complete destination structure for each country-year, not a subset of partners. The index was then computed as:

$$\mathrm{HH}I_{\mathrm{it}}=\sum_{j\in J_{\mathrm{it}}}{\left(100\;s_{\mathrm{ijt}}\right)}^{2}.$$

On a 0–10,000 scale, where higher values indicate greater concentration and, therefore, lower diversification. For descriptive interpretation, conventional thresholds were used: HHI<1000 (unconcentrated destination markets; greater diversification), 1000≤HHI≤1800 (moderate concentration), and HHI>1800 (high concentration; lower diversification). ^52^ Nevertheless, HHI interpretation was handled with methodological caution, since the index may present interpretive validity limitations if destination granularity changes or if small shares are truncated; therefore, the analysis prioritized temporal trajectories and destination-structure context over mechanical threshold-based judgments. ^53^ ^,^ ^54^

Revealed specialization was assessed using the Revealed Symmetric Comparative Advantage (RSCA), which transforms the classical Balassa Revealed Comparative Advantage (RCA) into a symmetric and bounded scale. First, RCA was calculated for country i , product p (HS 030743), and year t as:

$$\mathrm{RC}A_{\mathrm{it}}=\frac{X_{\mathrm{ip},t}/X_{i\cdot ,t}}{X_{\cdot p,t}/X_{\cdot \cdot ,t}}$$

where $X_{\mathrm{ip},t}$ is country i ’s exports of HS 030743; $X_{i\cdot ,t}$ is country i ’s total exports (all products); $X_{\cdot p,t}$ is world exports of HS 030743; and $X_{\cdot \cdot ,t}$ is total world exports. The conceptual basis of RCA follows the foundational revealed comparative advantage approach based on export patterns. ^55^

Subsequently, the reported and analyzed indicator was its symmetric version:

$$\mathrm{RSC}A_{\mathrm{it}}=\frac{\mathrm{RC}A_{\mathrm{it}}-1}{\mathrm{RC}A_{\mathrm{it}}+1}$$

This indicator ranges from [−1,1] and is centered at 0 as the neutral point. This transformation was adopted because it reduces the inherent asymmetry of RCA and facilitates descriptive intertemporal and cross-country comparisons while preserving direct interpretation: positive values indicate relative revealed specialization, whereas negative values indicate relative non-specialization. This choice is supported by the literature introducing and applying RSCA in empirical studies, ^56^ as well as later reviews recognizing RSCA as a symmetric alternative for measuring specialization. ^57^

Finally, after data cleaning and harmonization, a country-year panel was constructed including: (i) total export value of HS 030743, (ii) the number of destination countries with positive exports, (iii) $\mathrm{HH}I_{\mathrm{it}}$ , and (iv) $\mathrm{RSC}A_{\mathrm{it}}$ . This panel provided the basis for the comparative description of diversification and specialization trajectories across the six exporting countries over the 2020–2024 period.

### 2.1 Complementary descriptive and exploratory analyses

To make the comparison across countries and years explicit, the country–year panel was summarized with conventional descriptive statistics. For each country, the mean, standard deviation (SD), coefficient of variation (CV, in %) and range of the HHI and of total export value over 2020–2024 were computed, together with the absolute and relative change in HHI between 2020 and 2024 and the compound annual growth rate (CAGR) of export value. Two concentration ratios were added as intuitive complements to the HHI: CR1, the share of the leading destination in total exports, and CR3, the combined share of the three leading destinations; for Spain, reporter–partner self-flows were excluded from these ratios. The destination-level RSCA values reported in Tables 1–6 (five leading destinations per country) were summarized as the unweighted mean and SD across destinations and years, the range, and the percentage of destination–year observations with positive RSCA (revealed specialization).

Because the panel is small (six countries × five years = 30 country–year observations) and the indicators are bounded and non-normally distributed, exploratory comparisons relied on rank-based non-parametric procedures. Differences among countries in HHI, mean destination-level RSCA and export value were examined with the Kruskal–Wallis H test, ^58^ reporting the rank-based epsilon-squared (ε ^2^ = H/(n − 1)) as an effect size; ^59^ pairwise contrasts were screened with Mann–Whitney U tests under Holm’s sequential correction. ^60^ With five observations per country the smallest attainable pairwise p-value after correction is 0.119, so pairwise results are reported descriptively through mean ranks. Year-to-year change common to all countries was tested with the Friedman test, using countries as blocks, ^61^ and the 2020 and 2024 values were compared with the Wilcoxon signed-rank test. Monotonic associations between HHI, mean RSCA and export value were screened with Spearman’s rank correlation (ρ) on the 30 country–year observations and, as a robustness check, on the six country means. Given the repeated observations within countries, p-values are approximate and are interpreted as screening evidence rather than as confirmatory inference; two-sided tests with α = 0.05 were used, and all computations were performed in Python 3 with SciPy 1.17.

## 3. Results

### 3.1 China

China combined export volatility with a steady dispersion of its destination portfolio ( Table 1). Total exports fell by one third in 2023 (from USD 2,314 million to 1,559 million) and only partly recovered in 2024, yet the HHI declined every year, from 1,134 to 704 (−38%), crossing the conventional 1,000-point boundary between moderate and unconcentrated markets in 2021. The share of the leading destination halved (CR1 from 24% to 13%) and the three leading markets absorbed 34% of exports in 2024 compared with 50% in 2020. Diversification therefore proceeded through the contraction of sales to Thailand and Korea rather than through the loss of any single market, and the residual group of destinations grew from 41% to 47% of the total.

**Table 1.** China’s squid and cuttlefish exports (USD millions), market diversification of China’s squid and cuttlefish exports, and RSCA of China’s squid and cuttlefish exports.

Importers	2020	2021	2022	2023	2024
Japan	211	215	266	230	220
United States of America	103	191	186	144	168
Spain	64	92	90	81	151
Korea, Republic of	269	206	296	176	181
Thailand	457	337	371	168	178
Others	781	1051	1106	760	799
Total	1885	2091	2314	1559	1697
**Years**	**2020**	**2021**	**2022**	**2023**	**2024**
HHI of export shares (0–10,000)	1134	949	850	771	704
**Importers**	**2020**	**2021**	**2022**	**2023**	**2024**
Japan	0.34	0.35	0.41	0.52	0.51
United States of America	−0.52	−0.31	−0.34	−0.23	−0.20
Spain	0.52	0.61	0.54	0.63	0.77
Korea, Republic of	0.53	0.38	0.48	0.44	0.45
Thailand	0.85	0.77	0.76	0.66	0.63

Source: UN Comtrade ^47^; authors’ calculations.

The relevance of this pattern lies in what it implies for exposure: the decline in dependence on historically dominant buyers, rather than the opening of new markets, is what lowered concentration, and the broad residual destination base gives China more room to reallocate sales when a specific market weakens, as happened in 2023.

From a competitiveness perspective, RSCA reflects an interesting repositioning: China maintains positive comparative advantages in Japan, Spain, Korea, and Thailand, but with different trajectories. The advantage in Spain strengthens markedly, and Japan also shows a stronger RSCA, compatible with improved positioning in demanding markets. In contrast, Thailand remains positive but declines over time, in parallel with the lower weight of Thailand in China’s portfolio; whether this reflects stronger competition from other exporters cannot be established with these data. The United States is particularly relevant: RSCA remains negative throughout the period, but it becomes progressively less negative, which is consistent with a reduction in China’s comparative disadvantage in that market, even if it has not yet achieved a revealed advantage.

### 3.2 Indonesia

Indonesia is the mirror image of China: exports grew every year, from USD 425 million to 727 million (CAGR 14.4%), but the growth was absorbed by a shrinking set of buyers ( Table 2). The HHI stayed far above the 1,800-point threshold for high concentration in all years and rose from 2,964 to 3,894 (+31%); China alone took 54% of exports in 2024 and the three leading markets 91%, while the residual group of destinations fell from 10% to 5% of the total. Growth, in other words, deepened dependence rather than diluting it.

**Table 2.** Indonesia’s squid and cuttlefish exports (USD millions), market diversification of Indonesia’s squid and cuttlefish exports, and RSCA of Indonesia’s squid and cuttlefish exports.

Importers	2020	2021	2022	2023	2024
China	198	279	270	371	391
Viet Nam	92	95	184	142	224
Taipei, Chinese	74	55	51	57	46
Italy	10	12	24	14	16
Korea, Republic of	9	9	12	13	11
Others	42	43	46	30	39
Total	425	492	587	627	727
**Years**	**2020**	**2021**	**2022**	**2023**	**2024**
HHI of export shares (0–10,000)	2964	3735	3211	4114	3894
**Importers**	**2020**	**2021**	**2022**	**2023**	**2024**
China	0.41	0.42	0.34	0.40	0.39
Viet Nam	0.75	0.73	0.83	0.77	0.79
Taipei, Chinese	0.75	0.57	0.49	0.56	0.43
Italy	0.39	0.32	0.58	0.46	0.44
Korea, Republic of	−0.29	−0.36	−0.36	−0.31	−0.45

Source: UN Comtrade ^47^; authors’ calculations.

The main internal pattern is the consolidation of a China–Viet Nam axis as the core of Indonesia’s export growth. Over time, these two markets account for a rising share of total exports, while the “Others” category loses relative importance. This is consistent with Indonesia expanding successfully but with a less distributed commercial structure. Such a configuration may be efficient in the short run, but it leaves Indonesia exposed to any change in demand, trade conditions, or sanitary requirements in either of these two markets.

The RSCA pattern reinforces this interpretation. Indonesia shows positive and relatively stable comparative advantages in China and especially in Viet Nam, confirming strong integration into the Asian trade circuit. However, Taipei, Chinese shows a gradual weakening in RSCA, suggesting a loss of relative specialization in that destination. Korea, Republic of remains negative and worsens over time, indicating a persistent and deepening comparative disadvantage. In other words, Indonesia is not broadening competitiveness uniformly; instead, it is deepening a geographically concentrated specialization.

### 3.3 India

India shows the most stable destination structure in the sample ( Table 3). Exports rose from USD 468 million to 727 million in 2022 and then eased to 610 million, but the HHI moved only within a narrow band (1,260–1,405; CV = 4.4%, the lowest of the six countries), and the leading destination never exceeded 28% of the total. The decline in export value in 2023–2024 was therefore not accompanied by any reconfiguration of markets, which points to demand or price effects rather than to a loss of outlets, although this cannot be verified with customs values alone.

**Table 3.** India’s squid and cuttlefish exports (USD millions), market diversification of India’s squid and cuttlefish exports, and RSCA of India’s squid and cuttlefish exports.

Importers	2020	2021	2022	2023	2024
Spain	125	154	201	148	161
Italy	60	102	117	107	102
Thailand	74	66	98	110	92
China	51	60	47	64	46
United States of America	20	46	61	42	36
Others	139	173	204	194	174
Total	468	600	727	665	610
**Years**	**2020**	**2021**	**2022**	**2023**	**2024**
HHI of export shares (0–10,000)	1371	1318	1405	1260	1389
**Importers**	**2020**	**2021**	**2022**	**2023**	**2024**
Spain	0.92	0.92	0.93	0.91	0.92
Italy	0.78	0.79	0.79	0.78	0.79
Thailand	0.84	0.77	0.82	0.87	0.86
China	0.22	0.26	0.32	0.44	0.37
United States of America	−0.62	−0.41	−0.36	−0.47	−0.51

Source: UN Comtrade ^47^; authors’ calculations.

The strongest pattern in the table is the presence of a stable “triad” of markets (Spain, Italy, and Thailand), which supports India’s export base without creating overwhelming dependence on a single destination. Unlike Indonesia or Morocco, India does not rely excessively on one buyer. In addition, the “Others” category remains consistently relevant, serving as a buffer. This reflects a functional diversification pattern: not highly fragmented, but sufficiently distributed to reduce vulnerability.

In competitiveness terms, India exhibits one of the most consistent profiles in the sample. RSCA is high and persistently positive in Spain, Italy, and Thailand, indicating strong and stable specialization in those destinations. The case of China is also notable because RSCA improves over the period (despite some fluctuation at the end), which suggests a stronger relative position there. By contrast, the United States remains negative throughout the period; although export values are not negligible, India’s relative competitive position in that market has not reached revealed comparative advantage.

### 3.4 Spain

Spain operates on a different scale ( Table 4): its exports, USD 3,627 million in 2020 and 6,875 million in 2024 (CAGR 17.3%), exceed the combined value of the other five countries in every year. Scale, however, did not translate into dispersion. The HHI remained in the high-concentration zone throughout (2,619–3,426), China and the United States absorbed between 68% and 74% of the total, and the residual group of destinations did not exceed 18%. Spain’s portfolio is therefore large but narrow, and its year-to-year concentration tracked the swings in Chinese purchases.

**Table 4.** Spain’s squid and cuttlefish exports (USD millions), market diversification of Spain’s squid and cuttlefish exports, and RSCA of Spain’s squid and cuttlefish exports.

Importers	2020	2021	2022	2023	2024
China	1853	2263	4247	3865	3234
United States of America	654	1182	1510	1364	1463
Spain	242	332	399	383	361
Italy	140	174	231	266	326
France	184	280	269	200	247
Others	554	859	1115	1013	1244
Total	3627	5090	7769	7093	6875
**Years**	**2020**	**2021**	**2022**	**2023**	**2024**
HHI of export shares (0–10,000)	3037	2619	3426	3402	2747
**Importers**	**2020**	**2021**	**2022**	**2023**	**2024**
China	0.53	0.52	0.51	0.50	0.52
United States of America	−0.14	−0.02	−0.18	−0.11	0.02
Spain	0.43	0.40	0.28	0.33	0.28
Italy	0.21	0.24	0.27	0.30	0.37
France	0.58	0.58	0.52	0.45	0.49

Source: UN Comtrade ^47^; authors’ calculations.

The central pattern is the dominance of the China–United States pair, which explains a large share of total exports throughout the period. When China’s absorption rises, HHI increases (as in 2022–2023); when the relative weight of other destinations grows (including “Others” and Italy), concentration declines somewhat (as in 2024). Therefore, HHI dynamics in Spain co-vary with fluctuations in Chinese demand. This is consistent with a large-scale export structure, but one that remains sensitive to the distribution of sales across a few major destinations.

The RSCA results provide a more nuanced reading: Spain maintains positive comparative advantages in China and France, and shows a clear improvement in Italy, compatible with stronger positioning within the European/Mediterranean trade network. The United States is especially noteworthy because RSCA shifts from negative or near-neutral levels to slightly positive in 2024, a pattern compatible with improved relative competitiveness or with a more favorable export composition; the data do not allow the two to be separated. By contrast, the destination labeled Spain reflects reporter-partner self-flows in UN Comtrade, typically re-exports, re-imports, or coding mismatches. These rows are kept in the table for transparency but are not treated as external dependence; recomputing the indices without them does not change Spain’s concentration reading.

### 3.5 Morocco

Morocco has the most concentrated portfolio of the six countries ( Table 5). Exports grew from USD 312 million to 417 million with marked year-to-year swings (CV = 17.9%), but Spain and Italy jointly absorbed between 87% and 95% of the total, the HHI ranged from 4,177 to 6,053 (mean 5,058), and the residual group of destinations collapsed from 9% of exports in 2020 to 1% in 2024. Morocco’s growth is thus inseparable from the purchasing decisions of two Mediterranean buyers.

**Table 5.** Morocco’s squid and cuttlefish exports (USD millions), market diversification of Morocco’s squid and cuttlefish exports, and RSCA of Morocco’s squid and cuttlefish exports.

Importers	2020	2021	2022	2023	2024
Spain	177	255	238	310	272
Italy	95	124	53	113	126
Greece	4	3	3	6	6
Portugal	3	2	7	5	5
Japan	4	2	2	6	3
Others	29	19	11	28	5
Total	312	405	313	468	417
**Years**	**2020**	**2021**	**2022**	**2023**	**2024**
HHI of export shares (0–10,000)	4177	4904	6053	4980	5176
**Importers**	**2020**	**2021**	**2022**	**2023**	**2024**
Spain	0.41	0.49	0.59	0.49	0.49
Italy	0.75	0.76	0.58	0.64	0.71
Greece	0.68	0.56	0.54	0.62	0.66
Portugal	−0.22	−0.45	0.22	−0.19	−0.14
Japan	0.36	−0.10	−0.20	0.54	0.32

Source: UN Comtrade ^47^; authors’ calculations.

The most important internal pattern is that Spain functions as the anchor market, and changes in Spain’s purchases explain much of the variation in Morocco’s total exports. Italy acts as a second pillar, but with greater volatility. The “Others” category declines sharply toward the end of the period, implying an even less distributed market structure. This not only increases commercial risk but also reduces Morocco’s ability to offset downturns through alternative destinations.

In RSCA terms, Morocco maintains positive comparative advantages in Spain, Italy, and Greece, confirming strong competitiveness within the Mediterranean area. However, this competitiveness is geographically concentrated. Portugal shows an unstable pattern (mostly negative or near zero), and Japan shifts sign across the period, indicating a less consolidated position outside the core regional corridor. In other words, Morocco is competitive, but its competitiveness is embedded in a narrow regional network rather than spread across a broad global market base.

### 3.6 Argentina

Argentina is the most volatile case ( Table 6). In 2020 China took half of Argentine exports (CR1 = 50%) and the HHI stood at 2,875; the collapse of sales to China in 2021 (from USD 195 million to 60 million) cut the HHI to 1,546 and shifted market leadership to Korea, and the partial return of Chinese purchases in 2024 pushed the index back up to 1,783. Argentina’s HHI therefore shows the largest relative dispersion in the sample (CV = 31.1%), and its diversification is less a strategy than a by-product of the fluctuations of a single buyer.

**Table 6.** Argentina’s squid and cuttlefish exports (USD millions), market diversification of Argentina’s squid and cuttlefish exports, and RSCA of Argentina’s squid and cuttlefish exports.

Importers	2020	2021	2022	2023	2024
China	195	60	56	63	118
Thailand	26	26	55	42	30
Spain	40	27	30	24	35
Japan	14	9	12	19	15
Korea, Republic of	51	61	72	68	58
Others	63	63	69	72	96
Total	389	247	293	288	352
**Years**	**2020**	**2021**	**2022**	**2023**	**2024**
HHI of export shares (0–10,000)	2875	1546	1573	1516	1783
**Importers**	**2020**	**2021**	**2022**	**2023**	**2024**
China	0.68	0.51	0.36	0.48	0.64
Thailand	0.80	0.90	0.96	0.95	0.92
Spain	0.67	0.65	0.68	0.59	0.69
Japan	0.73	0.61	0.64	0.74	0.84
Korea, Republic of	0.82	0.84	0.83	0.86	0.83

Source: UN Comtrade ^47^; authors’ calculations.

The key internal pattern is the change in the leading market: China dominates in 2020, then Korea becomes the main destination in 2021–2023, and China returns to a leading role in 2024. This shift coincides with export volatility and with a redistribution of flows across markets. In addition, the increasing weight of “Others” toward the end of the period suggests that Argentina expanded its destination base, although not enough to prevent China’s rebound from increasing concentration again.

From the competitiveness perspective, Argentina shows a very favorable feature: RSCA is consistently high and positive in all reported markets (China, Thailand, Spain, Japan, and Korea). This implies that Argentina’s main challenge is not product competitiveness, but rather instability in the geographic allocation of export flows or shifts in external demand. The case of China is especially revealing: RSCA declines and then recovers, mirroring the loss and return of China’s market leadership. At the same time, Korea and Thailand maintain very high RSCA values, confirming that Argentina preserves strong specialization in Asia even when the leading buyer changes.

Figure 1 makes the contrast between profiles visible. China exhibits a more distributed portfolio in 2024, with a lower relative weight of the largest named destination and a broader combined participation of secondary destinations, which is consistent with a diversification pattern. Indonesia, in contrast, shows a stronger concentration in its top destinations by 2024, as the largest and second-largest named markets account for a greater share of total exports and the residual “Others” segment becomes less prominent. India maintains a comparatively stable portfolio composition across both years, reflecting a moderate and relatively balanced structure rather than sharp reconfiguration. Spain and Morocco remain highly concentrated, although with different profiles. Spain preserves a large dependence on its top destinations despite some redistribution among lower-ranked markets, while Morocco displays the most concentrated structure in the comparison, with an especially dominant top destination and a very limited residual share in 2024. Argentina presents an intermediate but dynamic pattern: the portfolio remains centered on a few leading destinations, yet the relative composition across ranked segments shifts between 2020 and 2024, suggesting a reallocation of export shares rather than a fully stable market structure. The value of this ranked view is that it shows where concentration comes from: a single dominant buyer in Morocco and Indonesia, a pair of buyers in Spain, and a rotating leader in Argentina, as opposed to the flat profile of China and India.

**Figure 1.**
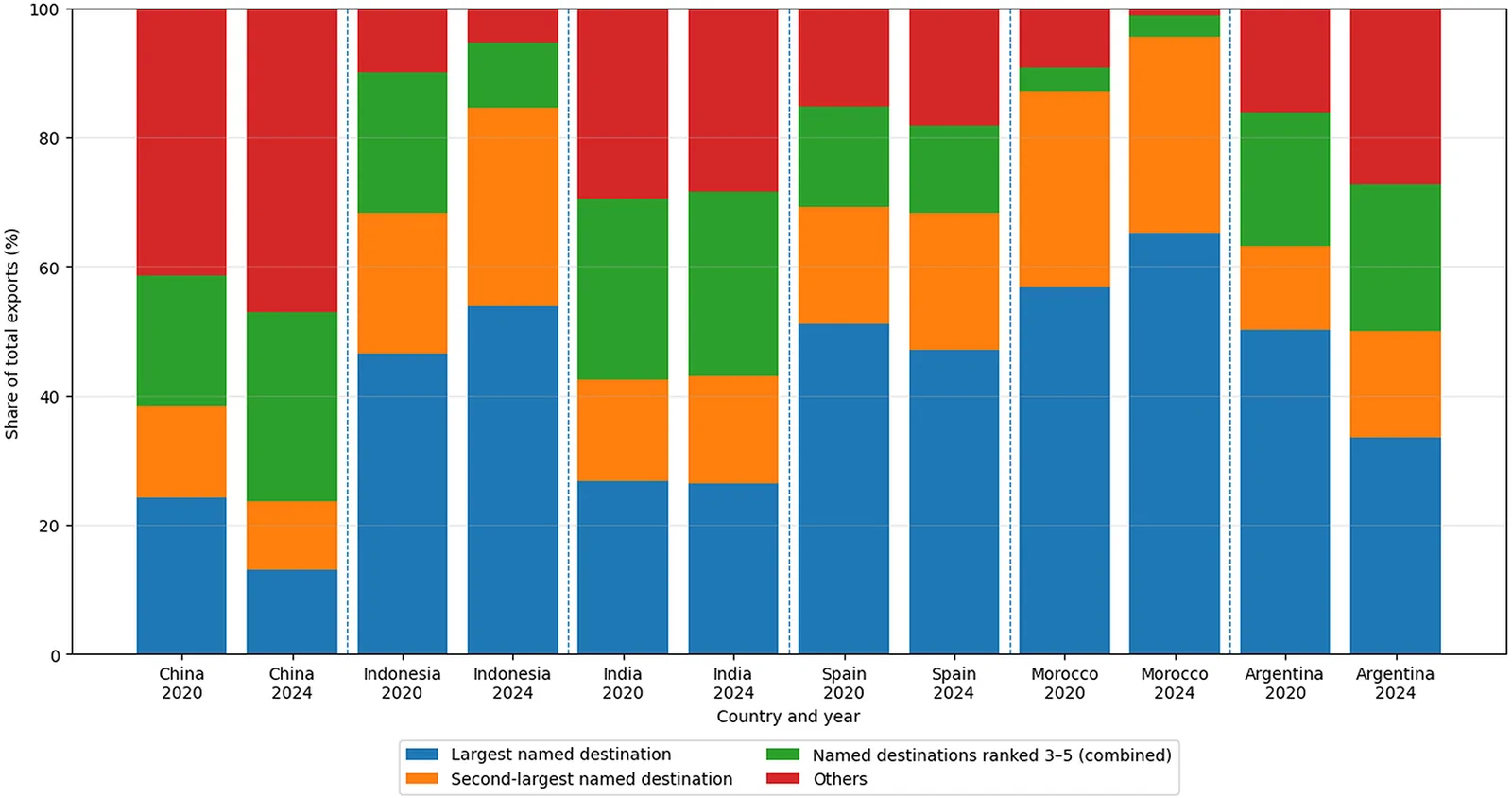
Comparative destination portfolio structure (Ranked shares), 2020 vs. 2024.

India and Argentina display the most stable and favorable RSCA profiles ( Figure 2), with consistently high and positive RSCA values across most reported destinations, indicating sustained relative export positioning over time. China also maintains positive RSCA values in key destinations (Japan, Spain, Korea, and Thailand), but with a clear internal reconfiguration: RSCA strengthens in Spain and remains solid in Japan, while it weakens in Thailand. In the United States, China’s RSCA remains negative in both years, although the shift toward zero suggests a reduction in relative disadvantage by 2024.

**Figure 2.**
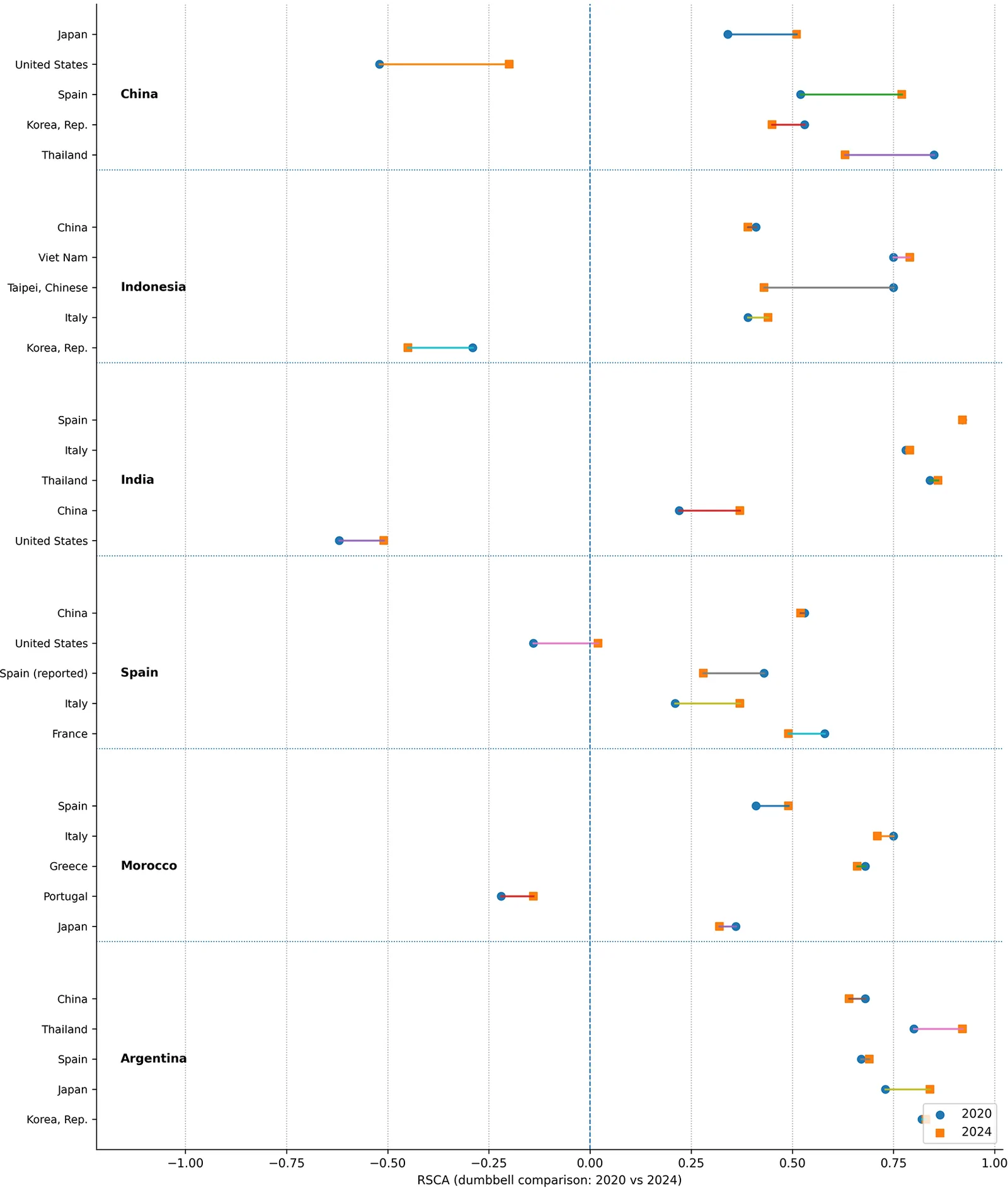
Changes in RSCA by destination market between 2020 and 2024 for HS 030743 exports: A Dumbbell plot.

The figure also highlights more selective or geographically concentrated specialization patterns. Indonesia retains positive RSCA in China, Viet Nam, Taipei (Chinese), and Italy, but the decline in Taipei and the persistently negative RSCA in Korea indicate that its competitive positioning is uneven across destinations. Spain presents a mixed profile, with positive RSCA in China, France, and Italy and a modest improvement in the United States (approaching neutrality in 2024), while destination-specific variation remains evident. Morocco maintains positive RSCA in its core Mediterranean destinations (Spain, Italy, and Greece), but with greater instability in Portugal and Japan. Read together with the HHI results, these patterns show that revealed positioning and destination concentration are distinct dimensions: a country can strengthen its position in a market on which it is becoming more dependent (Indonesia in China) or hold strong positions in markets whose weight is falling (Argentina in China in 2021–2023).

The most notable change between 2020 and 2024 is China’s deconcentration ( Figure 3), with HHI declining from 1134 in 2020 to 704 in 2024, which implies a transition from a moderately concentrated structure to an unconcentrated destination portfolio. India remains comparatively stable within the moderate-concentration range (1371 to 1389), indicating a relatively balanced export structure with limited net change over the period. Argentina shows a substantial decline in concentration (2875 to 1783), moving from high concentration toward the upper bound of the moderate range, which is consistent with a more distributed destination structure by 2024 despite partial reconcentration relative to 2021–2023.

**Figure 3.**
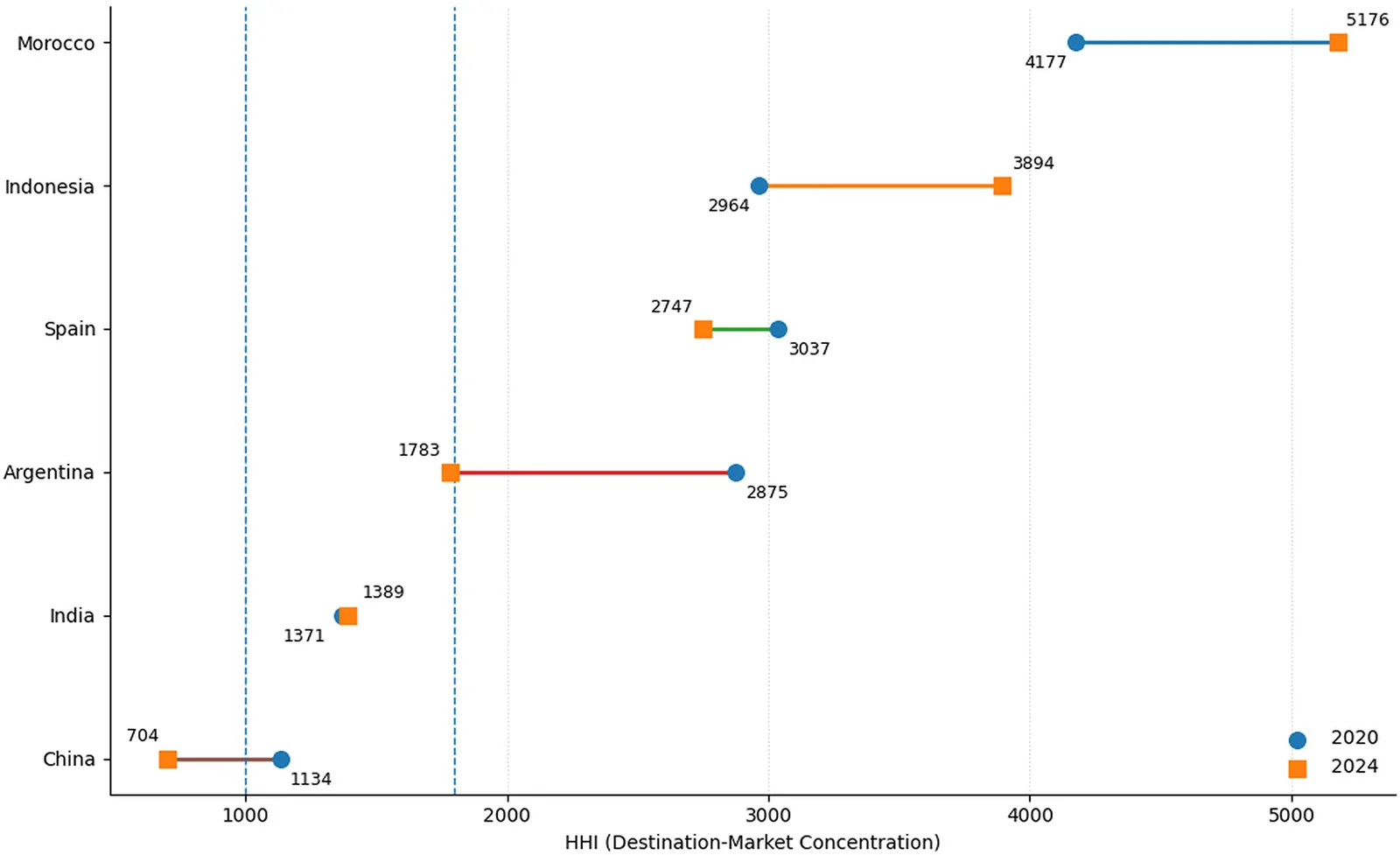
Changes in destination-market concentration (HHI) between 2020 and 2024 for HS 030743 Exports: A Dumbbell plot.

By contrast, Spain, Indonesia, and Morocco remain in the high-concentration zone in both years, although with different trajectories. Spain records a moderate reduction in HHI (3037 to 2747), suggesting some improvement in destination distribution, but concentration remains clearly high. Indonesia increases from 2964 to 3894, indicating a stronger dependence on a reduced set of destinations by 2024. Morocco also becomes more concentrated, rising from 4177 to 5176, and remains the most concentrated case in the sample. Destination diversification thus moved in opposite directions within the same product and period: three countries deconcentrated (China, Argentina and, to a lesser extent, Spain) while three concentrated further (Indonesia, Morocco and, marginally, India), which is why the summary statistics and exploratory tests in Section 3.7 detect large differences between countries but no common temporal trend.

### 3.7 Comparative summary statistics and exploratory tests

Table 7 condenses the six country profiles into comparable summary measures. The ranking of mean HHI over 2020–2024 places China (882), India (1,349) and Argentina (1,859) in the unconcentrated-to-moderate range and Spain (3,046), Indonesia (3,584) and Morocco (5,058) in the high-concentration range. The coefficient of variation separates stable from unstable structures: India’s HHI varied by only 4.4% around its mean, whereas Argentina’s varied by 31.1%, with China (19.1%), Indonesia (13.4%), Morocco (13.3%) and Spain (12.1%) in between. The concentration ratios tell the same story in more intuitive units: in 2024 the leading destination absorbed 13% of Chinese exports but 65% of Moroccan exports, and the three leading markets ranged from 34% (China) to 97% (Morocco). Export growth and concentration did not move together in any simple way: the two fastest-growing exporters, Spain (CAGR 17.3%) and Indonesia (14.4%), are both highly concentrated, while the two countries whose exports contracted, China (−2.6%) and Argentina (−2.5%), are the two that deconcentrated most (−38% in HHI in both cases).

**Table 7.** Summary statistics of destination concentration (HHI), concentration ratios and export value of HS 030743 by country, 2020–2024.

Country	HHI, mean (SD)	HHI CV (%)	HHI min–max	ΔHHI 2020–2024, points (%)	Export value, mean (SD), USD million	Export CAGR 2020–2024 (%)	CR1 2020 → 2024 (%)	CR3 2020 → 2024 (%)	“Others” 2020 → 2024 (%)
China	882 (168)	19.1	704–1,134	−430 (−37.9)	1,909 (302)	−2.6	24.2 → 13.0	49.7 → 34.1	41.4 → 47.1
Indonesia	3,584 (480)	13.4	2,964–4,114	+930 (+31.4)	572 (118)	14.4	46.6 → 53.8	85.6 → 90.9	9.9 → 5.4
India	1,349 (59)	4.4	1,260–1,405	+18 (+1.3)	614 (96)	6.9	26.7 → 26.4	55.3 → 58.2	29.7 → 28.5
Spain	3,046 (368)	12.1	2,619–3,426	−290 (−9.5)	6,091 (1,696)	17.3	51.1 → 47.0	74.2 → 73.1	15.3 → 18.1
Morocco	5,058 (673)	13.3	4,177–6,053	+999 (+23.9)	383 (69)	7.5	56.7 → 65.2	88.5 → 96.9	9.3 → 1.2
Argentina	1,859 (578)	31.1	1,516–2,875	−1,092 (−38.0)	314 (56)	−2.5	50.1 → 33.5	73.5 → 59.9	16.2 → 27.3

*Note:* HHI on a 0–10,000 scale; SD = standard deviation; CV = coefficient of variation; CAGR = compound annual growth rate of export value; CR1 and CR3 = share of the leading destination and of the three leading destinations in total exports, computed from the named destinations in Tables 1–6 (for Spain, reporter–partner self-flows excluded); “Others” = residual share of all remaining destinations.

Table 8 summarizes the destination-level RSCA values of Tables 1–6. Argentina stands out with a mean RSCA of 0.73 (SD 0.15) and positive values in 100% of destination–year observations, followed by India (0.48; 80% positive). China, Indonesia and Morocco cluster around 0.36–0.38, and Spain shows the lowest mean (0.32), reflecting near-neutral positioning in the United States. Between 2020 and 2024 the mean RSCA rose in China (0.34 to 0.43) and India (0.43 to 0.49), remained roughly stable in Spain (0.32 to 0.34), Morocco (0.40 to 0.41) and Argentina (0.74 to 0.78), and fell in Indonesia (0.40 to 0.32).

**Table 8.** Summary statistics of destination-level RSCA of HS 030743 exports by country, 2020–2024.

Country	Mean RSCA (SD)	Min–max	Positive RSCA (% of destination–years)	Mean RSCA 2020 → 2024	Destinations with RSCA > 0 in 2024 (of 5)
China	0.38 (0.39)	−0.52 to 0.85	80	0.34 → 0.43	4
Indonesia	0.36 (0.40)	−0.45 to 0.83	80	0.40 → 0.32	4
India	0.48 (0.53)	−0.62 to 0.93	80	0.43 → 0.49	4
Spain	0.32 (0.23)	−0.18 to 0.58	84	0.32 → 0.34	5
Morocco	0.36 (0.36)	−0.45 to 0.76	76	0.40 → 0.41	4
Argentina	0.73 (0.15)	0.36 to 0.96	100	0.74 → 0.78	5

*Note:* 25 destination–year observations per country (five leading destinations × five years), taken from Tables 1–6; RSCA ranges from −1 to 1, with positive values indicating revealed specialization.

The exploratory tests ( Table 9) confirm that the heterogeneity described above is statistically distinguishable from year-to-year noise. Countries differed strongly in HHI (Kruskal–Wallis H = 27.4, p < 0.001, ε ^2^ = 0.95), in mean destination-level RSCA (H = 22.8, p < 0.001, ε ^2^ = 0.79) and in export value (H = 26.7, p < 0.001, ε ^2^ = 0.92); mean ranks order the countries from China (least concentrated) through India, Argentina, Spain and Indonesia to Morocco (most concentrated). By contrast, no common temporal effect was detected: the Friedman test did not reject equality across years for HHI (χ ^2^ = 3.07, p = 0.547), mean RSCA (χ ^2^ = 4.40, p = 0.354) or export value (χ ^2^ = 6.00, p = 0.199), and the 2020 and 2024 HHI values did not differ systematically across the six countries (Wilcoxon p = 1.00), because three countries deconcentrated while three concentrated. Trajectories are therefore country-specific rather than shared. Across the 30 country–year observations, HHI was moderately and inversely associated with mean RSCA (Spearman ρ = −0.40, p = 0.030) and mean RSCA was inversely associated with export value (ρ = −0.58, p < 0.001), whereas HHI and export value were not significantly related (ρ = −0.27, p = 0.142). The first association weakens when computed on the six country means (ρ = −0.54, p = 0.266), which indicates that it is driven mainly by between-country differences: the three countries with dispersed portfolios (China, India and Argentina) also held the strongest positions in their leading markets, while the largest exporter (Spain) combines high concentration with the weakest destination-level RSCA. These associations are descriptive regularities in a small panel with repeated observations per country and do not identify a direction of influence.

**Table 9.** Exploratory non-parametric tests on the country–year panel (six countries × five years).

Test	Variable(s)	Statistic	p	Effect size/note
Kruskal–Wallis (6 countries)	HHI	H(5) = 27.42	< 0.001	ε ^2^ = 0.95
Kruskal–Wallis (6 countries)	Mean destination-level RSCA	H(5) = 22.84	< 0.001	ε ^2^ = 0.79
Kruskal–Wallis (6 countries)	Export value	H(5) = 26.74	< 0.001	ε ^2^ = 0.92
Pairwise Mann–Whitney, Holm-corrected	HHI, 15 country pairs	U = 0–25	≥ 0.119	Minimum attainable p after correction = 0.119 (n = 5 per country)
Friedman (5 years, countries as blocks)	HHI	χ ^2^(4) = 3.07	0.547	No common year effect
Friedman (5 years, countries as blocks)	Mean destination-level RSCA	χ ^2^(4) = 4.40	0.354	No common year effect
Friedman (5 years, countries as blocks)	Export value	χ ^2^(4) = 6.00	0.199	No common year effect
Wilcoxon signed-rank (n = 6)	HHI, 2020 vs. 2024	W = 10	1.000	Three countries increased, three decreased
Wilcoxon signed-rank (n = 6)	Mean RSCA, 2020 vs. 2024	W = 5	0.313	—
Spearman (n = 30 country–years)	HHI × mean RSCA	ρ = −0.40	0.030	ρ = −0.54 (p = 0.266) on the six country means
Spearman (n = 30 country–years)	HHI × export value	ρ = −0.27	0.142	—
Spearman (n = 30 country–years)	Mean RSCA × export value	ρ = −0.58	< 0.001	—
Spearman (n = 30 country–years)	HHI × CR1	ρ = 0.97	< 0.001	Consistency check between concentration measures

*Note:* ε ^2^ = rank-based epsilon-squared (H/(n − 1)); Friedman tests use the six countries as blocks; Spearman correlations are computed on the 30 country–year observations; p-values are two-sided and approximate given repeated observations within countries.

## 4. Discussion

The results describe a heterogeneous export landscape for HS 030743, where differences in destination structure are as relevant as differences in export scale. China and India present comparatively more balanced configurations, but through distinct trajectories: China moves toward lower concentration over time, whereas India maintains a relatively stable, moderately concentrated portfolio. This distinction is important in descriptive terms because it shows that diversification can emerge either through gradual deconcentration or through sustained structural balance, without implying a common underlying mechanism. By contrast, Indonesia, Spain, and Morocco remain persistently concentrated, although around different commercial cores (Asian corridor, China–United States axis, and Mediterranean markets, respectively), which is consistent with the broader literature showing that cephalopod trade is globally extensive but still shaped by dominant nodes and asymmetric market linkages. ^30^ ^,^ ^33^ Argentina, in turn, displays a more volatile pattern, with a visible reallocation of flows across Asian destinations and only partial stabilization by the end of the period, reinforcing the idea that destination structure itself is a meaningful descriptive indicator of commercial exposure. ^41^ The exploratory tests sharpen this reading: between-country differences in concentration are very large (ε ^2^ = 0.95) whereas no common year effect exists, so the heterogeneity is structural rather than a shared response to a particular year, such as the pandemic-affected 2020 baseline. ^62^

A second relevant finding is that the RSCA profiles add a distinct layer of interpretation that does not mechanically replicate HHI behavior. This supports the methodological decision of the study to treat diversification (HHI) and revealed specialization (RSCA) as parallel descriptive dimensions, rather than as variables linked by a causal hypothesis, in line with the literature on comparative advantage indicators and their symmetric transformation. ^9^ ^,^ ^56^ India and Argentina show the most stable positive RSCA patterns across their main destinations, suggesting strong revealed specialization even when their export values fluctuate. China, by contrast, shows a more dynamic repositioning process, with stronger relative performance in selected markets such as Spain and Japan, while gradually reducing its comparative disadvantage in the United States. Indonesia also exhibits positive RSCA in key destinations, but this competitiveness is concentrated in a narrow set of buyers, which means that strong specialization does not necessarily translate into broad market dispersion. Spain and Morocco similarly combine positive RSCA in core markets with concentrated destination structures, indicating that competitive strength can coexist with dependence on a limited number of export outlets.

The discussion of these patterns is especially relevant for sustainability-oriented interpretation, but the results should be read within the study’s strictly descriptive scope. As the literature on cephalopod supply chains emphasizes, trade performance alone does not capture governance quality, traceability integrity, or compliance conditions, all of which are central to sustainability assessments. ^15^ ^,^ ^16^ For this reason, the patterns observed in HHI and RSCA are interpreted here as descriptive signals of commercial resilience or vulnerability, not as evidence of sustainable or unsustainable fisheries outcomes. For example, lower concentration may indicate a more distributed destination portfolio and potentially lower dependence risk, but it does not by itself reveal whether the underlying supply chain is better governed. Likewise, strong RSCA values indicate relative export positioning, yet they may coexist with ecological variability or operational pressures that are common in squid and cuttlefish fisheries, where environmental fluctuations can affect supply conditions independently of market strategy. ^19^ ^,^ ^24^ This is precisely why the present analysis remains focused on characterization rather than causal attribution.

How do these results compare with previous studies of cephalopod trade? The network analyses of global cephalopod trade ^30^ ^,^ ^33^ identified a small group of countries—Spain and China among them—as the main hubs and described a market dominated by a few Asian and Mediterranean nodes, a structure that mirrors the hub-dominated architecture of seafood trade networks in general. ^63^ Our country-level results are consistent with that architecture but add a within-country reading of dependence, and Spain illustrates why the two readings differ. In the network literature Spain appears as a central importer, processor and re-exporter of cephalopods, ^30^ ^,^ ^64^ and the very large export values in Table 4, together with the self-flows and the moderate destination-level RSCA, are consistent with an entrepôt role in which part of the exported product originates from imports rather than from domestic catches. High concentration in a hub of this kind reflects the geography of demand (China and the United States) more than a lack of commercial capacity, which is the opposite of what a similar HHI value means for Morocco, whose concentration reflects a single regional outlet. Comparable index values can thus carry different substantive meanings, a point that concentration-based diagnoses of export vulnerability ^41^ ^,^ ^65^ tend to underplay and that the joint reading of HHI, concentration ratios and RSCA helps to surface.

The Chinese and Indonesian trajectories can be read against the literature on Asian demand. China’s changing seafood system, with rising domestic consumption, a large processing-and-re-export industry and an expanding distant-water squid fleet, ^4^ ^,^ ^19^ ^,^ ^66^ in a context of rising global cephalopod catches, ^67^ is compatible with both of our observations: as an exporter, China is dispersing its sales across a broader set of markets, while as an importer it has become the anchor buyer for Indonesia (54% of exports in 2024) and, intermittently, for Argentina (50% in 2020, 34% in 2024). Indonesia’s deepening dependence on the China–Viet Nam axis is consistent with the growing weight of intermediate flows toward regional processing hubs documented for aquatic-food trade as a whole, ^68^ which helps to explain why strong bilateral RSCA values coexist with a shrinking residual destination base and a worsening position in Korea. Our data cannot establish whether this is a deliberate specialization or the outcome of buyer power, but the combination of rapid growth, rising HHI and falling mean RSCA is the configuration that the export-diversification literature associates with the greatest exposure to demand shocks. ^65^ ^,^ ^69^

India and Argentina, the two countries with the strongest and most stable RSCA profiles, differ sharply in the stability of their destination structures, and the difference is instructive. India’s narrow HHI band (CV = 4.4%) and its triad of European and Thai buyers are consistent with the long-standing position of Indian marine-product exports in the European market described in the national export literature, ^7^ ^,^ ^8^ and with the general finding that more diversified portfolios dampen the transmission of external shocks. ^65^ ^,^ ^69^ Argentina’s volatility, by contrast, is the trade-side reflection of the biology and geopolitics of *Illex argentinus*: the species completes its life cycle in one year and its recruitment varies strongly with oceanographic conditions, ^35^ ^,^ ^70^ and the resource is harvested both by the national fleet and by distant-water fleets operating at the edge of the exclusive economic zone. ^4^ ^,^ ^27^ The abrupt fall of sales to China in 2021 and their partial recovery in 2024 are therefore more plausibly linked to supply and to the reallocation of Chinese purchases than to any change in Argentine competitiveness, which the uniformly high RSCA values suggest remained intact. This is a case in which the two indicators diverge—stable specialization, unstable destinations—and in which reading either one in isolation would be misleading.

Morocco’s extreme concentration also has an identifiable structural background. Seafood trade generally flows from developing exporters toward high-income consumer markets, ^71^ and Morocco’s cephalopod fisheries follow this pattern: they are oriented toward the European market, where Spain and Italy are the main consumers of these products. ^5^ ^,^ ^64^ Access to those markets is embedded in the institutional arrangements between Morocco and the European Union that the political-economy literature on the Saharan fishing sector has examined critically. ^6^ The collapse of the residual destination group from 9% to 1% of exports in 2024 means that Morocco has practically no alternative outlets, so any regulatory, sanitary or political disruption of the Mediterranean channel would translate almost directly into export losses. The contrast with the octopus fisheries of the same region, where declining yields have already been documented, ^36^ ^,^ ^72^ suggests that supply-side pressure and demand-side dependence may reinforce each other, although our data speak only to the latter.

The exploratory associations deserve a cautious but explicit interpretation. The inverse relation between HHI and destination-level RSCA (ρ = −0.40) and the inverse relation between RSCA and export scale (ρ = −0.58) both arise mainly from between-country differences, and both are consistent with a known property of Balassa-type indices: because RCA normalizes by the size of the exporter’s total trade, small exporters that are strongly specialized in a product obtain high values, whereas large, diversified economies obtain values close to the neutral point even when their absolute exports are the largest in the sample. ^9^ ^,^ ^73^ Spain and Argentina occupy the two extremes of this contrast. The associations should therefore not be read as evidence that diversification “causes” competitiveness or the reverse; they show that, in this product and period, the strength of revealed specialization scaled inversely with export size, and that the three countries with dispersed portfolios also held the strongest bilateral positions—precisely the configuration in which the sustainability literature expects trade concentration to be a meaningful risk signal. ^41^ ^,^ ^69^

Finally, the period itself calls for caution. The 2020 baseline coincides with the pandemic-related disruption of seafood logistics and food-service demand, ^62^ so part of the 2020–2021 change—most visibly the fall in China’s purchases from Argentina and the reordering of Spain’s markets—may reflect a return to pre-pandemic patterns rather than a structural shift. Five annual observations per country are sufficient to describe trajectories and to detect large between-country differences, as the effect sizes show, but not to separate cyclical from structural change; longer series extending back to the pre-pandemic years, and mirror statistics that reconcile exporter and importer reports, would be needed for that purpose. ^49^ ^,^ ^51^ Within these limits, the joint evidence from the summary statistics, the concentration ratios and the exploratory tests supports the three-profile typology proposed below, while making explicit that the profiles differ in kind and not only in degree.

## 5. Conclusions

In conclusion, the study identifies three broad descriptive export configurations for frozen squid and cuttlefish: a comparatively diversified and more resilient profile (China, and in a stable form India), a growth-oriented but concentrated profile (Indonesia and Spain), and a specialized yet structurally vulnerable profile due to destination dependence (Morocco, with Argentina showing partial vulnerability through market reorientation volatility). This typology extends the trade-network and specialization literature by offering a replicable cross-country mapping of destination concentration and relative export positioning based on reported customs statistics. The summary statistics and exploratory tests show that the profiles differ markedly between countries (ε ^2^ = 0.95 for HHI) while no common temporal trend exists over 2020–2024, and that dispersed portfolios tended to coincide with stronger bilateral positioning—a regularity that is descriptive, not causal. At the same time, and consistent with the study’s non-causal design, these findings should be understood as a baseline for further research rather than as explanatory evidence. Future work may build on this descriptive framework by integrating trade indicators with traceability, governance, and ecological variables to produce a more comprehensive sustainability assessment of cephalopod export systems.

Based on the descriptive evidence, the recommended action is to tailor trade monitoring and export strategy to each country’s destination structure rather than applying a single policy logic to all cases. Countries with broader or improving diversification (especially China, and in a more stable form India) should prioritize preserving destination breadth while strengthening traceability and compliance across markets, because diversification improves commercial resilience but does not, by itself, demonstrate sustainability performance. Countries with persistent concentration despite export growth (especially Indonesia and Spain) should gradually expand their destination portfolio to reduce dependence on a small number of anchor buyers, while countries with strong specialization but narrow destination dependence (especially Morocco, and partially Argentina) should prioritize risk management by maintaining core markets and developing secondary outlets. In all cases, HHI and RSCA should be monitored jointly as complementary descriptive indicators of market structure and relative export positioning, without treating either indicator as evidence of causality or as a substitute for governance and sustainability assessment.

Given the study’s strictly descriptive and non-causal design, future research should treat these results as a baseline for extension rather than as evidence of determinants. The main recommendation is to integrate customs-based indicators (HHI and RSCA) with variables that capture dimensions absent from trade records (especially traceability, governance quality, labor conditions, and ecological variability) so that sustainability can be assessed more comprehensively. In methodological terms, future studies should also strengthen robustness by using mirror statistics, sensitivity checks for destination granularity, longer time series that include the pre-pandemic years, and (where possible) greater product disaggregation beyond HS 030743 to distinguish species and processing profiles; with longer panels, the exploratory comparisons reported here could be replaced by panel models that separate country-specific from period-specific variation. Network-based trade indicators may also be added to refine structural interpretation, but they should be used under the same caution maintained in this study: they enhance descriptive mapping and comparative diagnosis, but they do not establish causal relationships.

## Institutional review board statement

Not applicable.

## Informed consent statement

Not applicable.

## Data Availability

Figshare: Mapping export sustainability in frozen cephalopods: destination concentration and revealed comparative advantage in China, Indonesia, India, Spain, Morocco, and Argentina (2020–2024). https://doi.org/10.6084/m9.figshare.32441517 ^74^ This project contains the following underlying data:
- Country–year export records for HS 030743 (frozen cuttlefish and squid) covering 2020–2024, organized by reporter country, importing partner, year, Harmonized System code, and export value (free on board, in United States dollars), derived from the United Nations International Trade Statistics Database (UN Comtrade). - Computed Herfindahl–Hirschman Index (HHI) values per country and year, including the destination shares used in their calculation. - Computed Revealed Symmetric Comparative Advantage (RSCA) values per country, destination market, and year. - Underlying values for all reported tables ( Tables 1– 6) and figures ( Figures 1– 3) of the manuscript. No human-subject data are included. Data are available under the terms of the Creative Commons Attribution 4.0 International license (CC-BY 4.0).
