# Supplementary material for: Mapping Export Sustainability in Frozen Cephalopods: Destination Concentration and Revealed Comparative Advantage in China, Indonesia, India, Spain, Morocco, and Argentina (2020–2024)
Source: F1000Res. 2026 Oct 8;15:971. Originally published 2026 Jun 18. [Version 2] doi: 10.12688/f1000research.183651.2 (PMC13653100; doi:10.12688/f1000research.183651.2)
Supplement: Peer review history

# Reviewer report: Reviewer response for version 2

**DOI:** 10.5256/f1000research.210283.r528244

**Published:** 2026-10-09

**Authors:** Antony Cristhian Gonzales-Alvarado

**Affiliations:** 1 Universidade Federal do ABC, São Bernardo do Campo, Brazil

I have read the manuscript again and consider that the comments and suggestions made in the previous review have been adequately addressed. The manuscript has improved, and in its current version, I consider it to be in good condition for INDEXING.

Is the work clearly and accurately presented and does it cite the current literature?

Partly

If applicable, is the statistical analysis and its interpretation appropriate?

Partly

Are all the source data underlying the results available to ensure full reproducibility?

Partly

Is the study design appropriate and is the work technically sound?

Partly

Are the conclusions drawn adequately supported by the results?

Partly

Are sufficient details of methods and analysis provided to allow replication by others?

Yes

Reviewer Expertise:

Biostatistics, Applied Statistics, Multivariate Data Analysis, Econometrics, Agricultural Economics, Environmental Economics, Scientific Research Methodology, and Bibliometric Analysis.

I confirm that I have read this submission and believe that I have an appropriate level of expertise to confirm that it is of an acceptable scientific standard.

# Reviewer report: Reviewer response for version 1

**DOI:** 10.5256/f1000research.202710.r506979

**Published:** 2026-09-02

**Authors:** Kurichithara K Sajikumar

**Affiliations:** 1 ICAR-Central Marine Fisheries Research Institute, Kochi, Kerala, India

The manuscript presents a meaningful and informative descriptive assessment of cephalopod export profiles across six important countries; China, Indonesia, India, Spain, Morocco, and Argentina. The selection of these countries provides a useful basis for cross-national comparison and contributes valuable baseline information on the geographical patterns and dynamics of the global cephalopod trade. However, the manuscript would benefit from strengthening its methodological framework, statistical analysis, and interpretation to enhance its scientific rigor and overall impact.

 Major Comments

**1. Methods section requires further clarification and improvement**

 The Methods section should be expanded to provide sufficient detail on the data sources, selection criteria, temporal coverage, variables analysed, and procedures used to calculate the different trade indicators. The rationale for selecting the six countries should also be clearly stated. 

 I think the method section has room for improvement

 Overall, the manuscript has potential to make a useful contribution to understanding the global cephalopod trade, but substantial methodological and analytical improvements are recommended before it can be considered for indexing.

Is the work clearly and accurately presented and does it cite the current literature?

Yes

If applicable, is the statistical analysis and its interpretation appropriate?

Partly

Are all the source data underlying the results available to ensure full reproducibility?

Yes

Is the study design appropriate and is the work technically sound?

Yes

Are the conclusions drawn adequately supported by the results?

Yes

Are sufficient details of methods and analysis provided to allow replication by others?

Yes

Reviewer Expertise:

Cephalopod biology, taxonomy, biodiversity, fisheries and fishery management, with particular emphasis on the systematics, species identification, distribution, ecology, population biology, and age and growth studies of cephalopods.

I confirm that I have read this submission and believe that I have an appropriate level of expertise to confirm that it is of an acceptable scientific standard.

# Reviewer report: Reviewer response for version 1

**DOI:** 10.5256/f1000research.202710.r495787

**Published:** 2026-07-08

**Authors:** Antony Cristhian Gonzales-Alvarado

**Affiliations:** 1 Universidade Federal do ABC, São Bernardo do Campo, Brazil

The manuscript addresses an interesting and relevant topic and has the potential to contribute to the field. However, I believe that substantial revisions are necessary before it can be considered for indexing.

 First, the analysis of the results could be strengthened by incorporating more robust statistical analyses. Although the study presents descriptive data, it would be beneficial to include statistical analyses that allow for the evaluation of differences among the variables examined. If the nature of the data does not permit inferential statistics, the authors should at least provide more comprehensive descriptive statistics (e.g., percentages, means, standard deviations, or other appropriate summary measures) to facilitate a clearer interpretation of the findings. In addition, exploratory analyses, such as correlation analyses or other suitable approaches, could provide valuable insights into potential relationships, patterns, or associations among the variables.

 Overall, the manuscript exhibits writing characteristics that suggest extensive reliance on artificial intelligence-assisted writing tools. Regardless of whether such tools were used, the manuscript requires careful language revision to improve its readability, coherence, and scientific style. Several sections read mechanically and lack the natural flow expected in a scientific manuscript.

 The Results section, in particular, requires a more in-depth interpretation. In many instances, the authors merely describe what is shown in the figures (e.g., "Figure X shows...") without explaining the scientific significance of the findings. Rather than simply restating the figures, the authors should provide a coherent narrative that highlights the key results and their relevance to the objectives of the study.

 Similarly, the Discussion is one of the weakest sections of the manuscript and requires substantial improvement. The comparison with previous studies is limited and, in some cases, superficial. I recommend expanding this section by incorporating a broader range of recent literature and providing a more critical discussion of similarities, differences, and the possible explanations for the observed findings. A stronger and more analytical discussion would significantly enhance the scientific value of the manuscript.

 Finally, I believe that the journal should maintain high scientific standards by indexing manuscripts that are methodologically rigorous and well written. While this study has scientific potential and addresses a relevant topic, it still requires major revisions regarding the statistical analyses, interpretation of the results, depth of the discussion, and overall writing quality before it can be considered for acceptance.

Is the work clearly and accurately presented and does it cite the current literature?

Partly

If applicable, is the statistical analysis and its interpretation appropriate?

Partly

Are all the source data underlying the results available to ensure full reproducibility?

Partly

Is the study design appropriate and is the work technically sound?

Partly

Are the conclusions drawn adequately supported by the results?

Partly

Are sufficient details of methods and analysis provided to allow replication by others?

Yes

Reviewer Expertise:

Biostatistics, Applied Statistics, Multivariate Data Analysis, Econometrics, Agricultural Economics, Environmental Economics, Scientific Research Methodology, and Bibliometric Analysis.

I confirm that I have read this submission and believe that I have an appropriate level of expertise to confirm that it is of an acceptable scientific standard, however I have significant reservations, as outlined above.
